# A short antimicrobial peptides family demonstrates efficacy to infection via a multimodal mechanism of action

**DOI:** 10.1128/aac.01343-25

**Published:** 2025-12-23

**Authors:** Yifan Liu, Pengfei Cui, Jingyi Sun, Shaoguo Ru

**Affiliations:** 1 Lab of Environmental Health and Ecological Engineering, College of Marine Life Science, Ocean University of China 12591, , Qingdao, China; 2 Jiangsu Medical College 117898 , Yancheng, China; 3 North China Sea Marine Forecasting Center of State Oceanic Administration, Qingdao, China; Columbia University Irving Medical Center, New York, New York, USA

**Keywords:** short antimicrobial peptides (SAMPs), multidrug-resistant (MDR), database-filtering technology, multimodal antimicrobial mechanism, sepsis infections

## Abstract

The escalating threat posed by multidrug-resistant (MDR) Gram-negative “superbugs” has intensified. Short antimicrobial peptides (SAMPs) have emerged as promising therapeutics with sustained potency and cost-effectiveness against drug-resistant infections. Here, we report a family of 15-residue SAMPs derived through modifying related amino acids of Kassporin-KS1 (FA), utilizing database-filtering technology to identify the most probable structural parameters related to Gram-negative bacteria. Most SAMPs exhibit sub-μM antimicrobial activity with reliable stability and low toxicity. Notably, KR and RK demonstrate significant efficacy in combating biofilms and sepsis infections *in vivo*. Furthermore, the acquisition of resistance by strains to SAMPs was not observed, primarily due to the multimodal antimicrobial mechanisms of SAMPs. We revealed that the multimodal mechanisms of SAMPs encompass unregulated membrane destabilization, induction of apoptotic-like death pathway, and interference with normal physiological processes. Overall, the rational design strategies proposed herein can be implemented to develop potent antimicrobial agents targeting MDR bacteria.

## INTRODUCTION

The escalating incidence of bacterial multidrug resistance (MDR) to current antibiotics poses a significant challenge to worldwide public health (1–3). Gram-negative bacteria are armed by an outer membrane that serves as a formidable impermeable barrier (4) and exhibit a propensity to form biofilm structures in the environment, rendering them intrinsically resistant to numerous hydrophobic antibiotics (5, 6). Infectious diseases driven by antimicrobial resistance (AMR) in healthcare settings, especially those caused by multidrug-resistant (MDR) Gram-negative bacteria with *Escherichia coli* as an example, are responsible for nearly 20% of deaths worldwide (7, 8). While in the latter stages of the disease, the formation of biofilms increases the resistance to antibiotics more than 10–1,000 times compared with planktonic cells, which makes the treatment of infections more difficult (9). Current therapy for infection is restricted to antibiotics, and only a small improvement in patient outcomes in sepsis infections (8, 10). Despite this, the progress in developing effective drug candidates against Gram-negative bacteria and biofilms remains inadequate (2, 11). Hence, there is a pressing need to innovate novel antimicrobial agents targeting MDR Gram-negative bacteria to treat infection with diverse modes of action.

Antimicrobial peptides (AMPs) are increasingly recognized as potential alternatives to traditional antibiotics for combating infections caused by resistant pathogens and biofilms (12). In addition to their effectiveness against free-floating bacteria, AMPs are hailed as promising therapeutic options for treating biofilm-related infections (13). The physical disruption of microbial membranes by AMPs through electrostatic interactions (14) and the diverse intracellular targets of AMPs (15) increase the likelihood of bacteria evading short-term resistance to AMPs (3), which is critical for the treatment of infection. Nevertheless, some challenges, such as limited stability, high toxicity to mammalian cells, and high cost, have hindered the clinical success of natural AMPs (16). Recently, comprehensive strategies employed to modify or engineer AMPs were developed, including optimizing AMPs through truncation and amino acid substitution utilizing natural AMP models (17), or designing AMPs from scratch (18–20). Database-filtering technology has been lauded as the most rational approach to design AMPs, as it allows for the identification of the most probable parameters from a set of AMPs utilizing the Antimicrobial Peptides Database (APD) to obtain optimal functional antimicrobial agents (21). Furthermore, the straightforward amino acid composition of short AMPs (SAMPs) enables easy customization based on specific requirements for antibacterial activity, toxicity, and stability, making them cost-effective and easily producible (22). Hence, our objective is to create shorter SAMPs (≤15 amino acids), emphasizing the structural characteristics and amino acid sequences that contribute to their desired properties. These modified methods will provide a template for the development of alternative antibacterial and antibiofilm alternatives.

**Fig 1:**
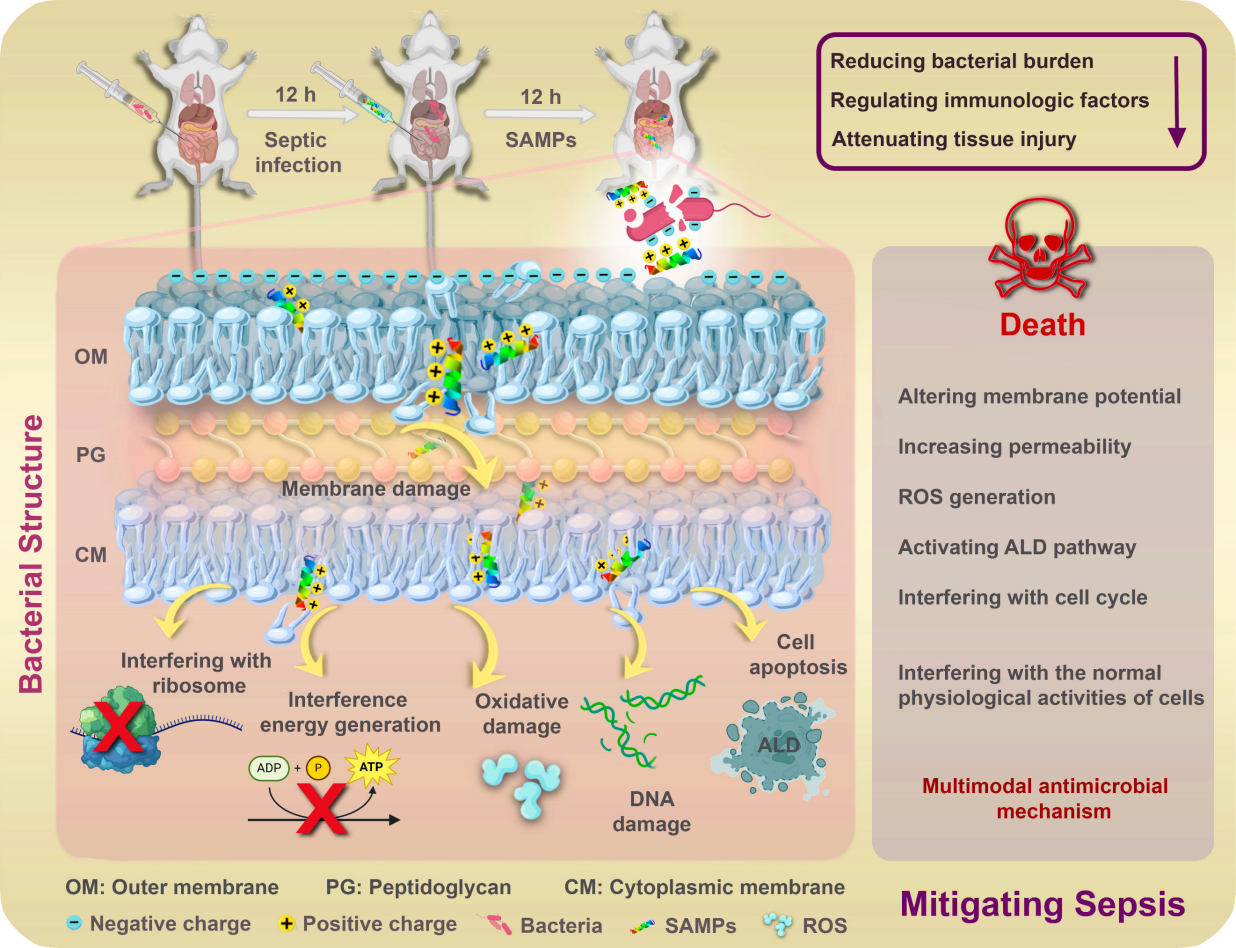
Schematic diagram of SAMPs-related sepsis treatment and antimicrobial mechanism.

In this research, database-filtering techniques were employed to determine the optimal structural parameters against Gram-negative bacteria of 116 shorter AMPs (10–20 amino acids), including peptide length, amino acid composition, net charge, and hydrophobic content, which were then used as guidelines for the modification of natural AMPs. Based on these optimal structural parameters, a natural peptide Kassporin-KS1 (abbreviated as FA, sequence FLALALIQEAIAKLK) with a specific length that lacks antibacterial activity against Gram-negative bacteria was extensively modified to improve its antibacterial activity. In light of the potential for AMR to develop in natural AMPs with high sequence homology to host defense peptides (23), the less common amino acid Tryptophan (W) in natural AMPs, known for its affinity for membrane interfaces (24), was strategically inserted twice into the peptide sequence to disrupt the sequence homology to natural sequences. Ultimately, a novel peptide family consisting of six novel synthetic SAMPs was successfully obtained by a straightforward approach of leveraging the filtered effective composition to modify distribution: (i) A natural peptide abundant in leucine (L) was selected from the APD based on the optimal length of 15 amino acids; (ii) The high-frequency amino acids L, glycine (G), lysine (K), and arginine (R) were then utilized to substitute the amino acids of the original peptide FA; (iii) The natural sequences were disrupted by the introduction of two hydrophobic residues W with strong membrane affinity; (iv) A net charge number of +5 or +6; (v) An appropriate hydrophobic content at 60%; (vi) The different amino acids optimized combination (GK, GR, KK, RR, RK, and KR) in SAMPs sequences; and (vii) All SAMPs were amidated to increase their stability. Subsequently, we evaluated the antibacterial activity, antibiofilm activity, and *in vivo* anti-inflammatory activity of the designed SAMPs family, verified the reliable stability and safety, and the multiple combined antibacterial mechanisms were also preliminarily explored (Fig. 1). The primary objective of this research was to establish a successful framework for the design of synthetic SAMPs, create concise yet potent SAMPs, and propose cost-effective alternatives to traditional antibiotics.

## RESULTS AND DISCUSSION

### Peptide design and characterization

**Fig 2:**
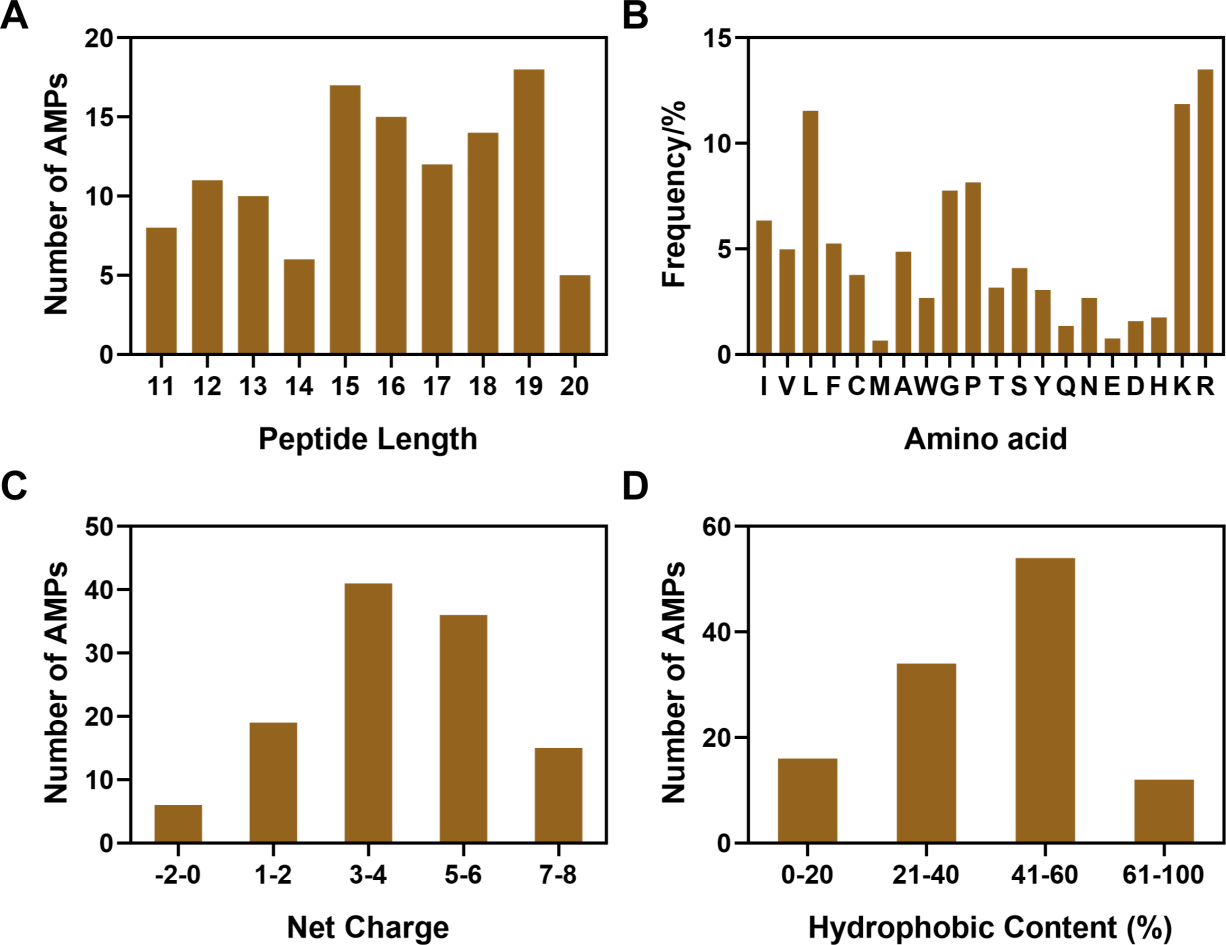
Structural parameter statistics of anti-Gram-negative AMPs of (**A**) peptide length, (**B**) frequency of amino acids, (**C**) net charge, and (**D**) hydrophobic content using the APD.

The identification results of 116 AMPs, which are exclusively effective against Gram-negative bacteria, from the APD (http://aps.unmc.edu/AP) are shown in Fig. 1. Based on the top three peptide lengths where the highest frequency occurs, the shortest length of 15 was selected to minimize expenses (Fig. 2A). The amino acid analysis in Fig. 2B revealed that the amino acids R, K, L, G, and proline (P) were the most frequently occurring amino acids. In our design, we opted for the more flexible G-amino acid over the rigid P-amino acid. Additionally, L-amino acid was used as a hydrophobic residue, and K-, R-amino acids were utilized to enhance positive charge. To assess the impact of K- and R-amino acids, we created amino acid pairs of GK, GR, KK, RR, RK, and KR to substitute the natural sequences and evaluate their antimicrobial efficacy. Meanwhile, the largest net charges of the most effective anti-Gram-negative bacteria peptides ranged from +5 to +6, with a preferred hydrophobic ratio of 40%–60% (Fig. 2C and D). Consequently, we obtained a series of ideal 15-residue length sequences with net charges of +5 or +6 and hydrophobic contents of 60%, which were designed by amino acid substitution of a 15-amino acid natural peptide FA (FLALALIQEAIAKLK) to acquire anti-Gram-negative bacteria activity. To be specific, the amino acids I7 and A12 in FA were replaced with W, and the amino acids A10 and I11 were replaced with L to improve the hydrophobicity. K and R were used to sequentially replace the uncharged amino acids A3 and A5 in the FA to enhance the overall net charge. Subsequently, the Q8 and E9 were replaced by GK, GR, KK, RR, RK, or KR to generate six novel SAMPs as outlined in Table 1.

**TABLE 1:** Sequences and relevant property parameters of the designed SAMPs

SAMPs	Sequence	Purity (%)	M. Wt. (actual value)	M. Wt. (theoretical value)	Net charge	Hydrophobic ratio（%）
FA	FLALALIQEAIAKLK	95.92	1641.04	1642.06	2	73
GK	FLKLRLWGKLLWKLK	95.34	1941.49	1942.51	5	60
GR	FLKLRLWGRLLWKLK	96.68	1969.5	1970.52	5	60
KK	FLKLRLWKKLLWKLK	95.13	2012.61	2013.63	6	60
RR	FLKLRLWRRLLWKLK	95.25	2068.64	2069.66	6	60
RK	FLKLRLWRKLLWKLK	95.21	2040.63	2041.65	6	60
KR	FLKLRLWKRLLWKLK	95.43	2040.63	2041.65	6	60

**Fig 3:**
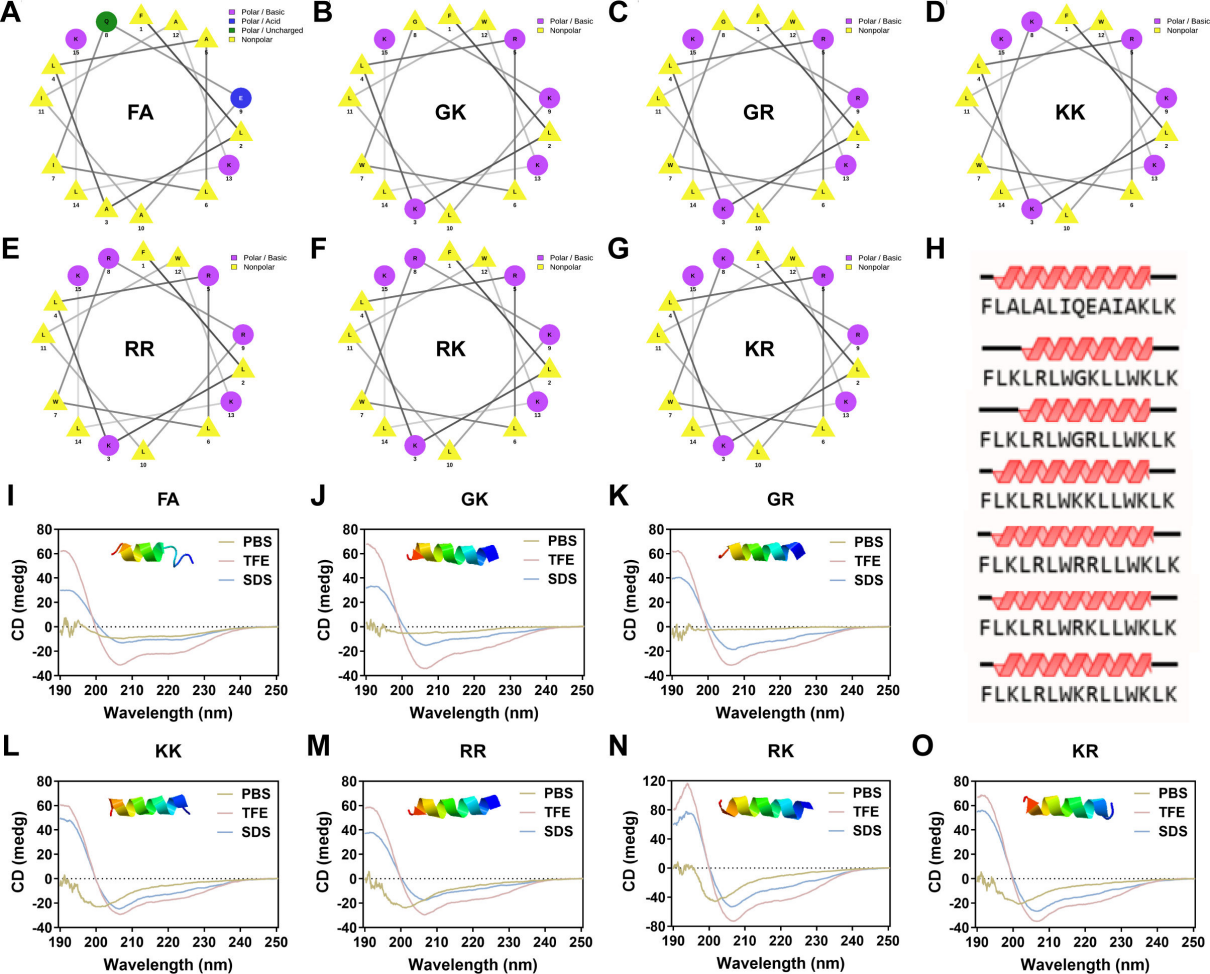
Structure characterization of SAMPs. Helical wheel projections of (**A**) FA, (**B**) GK, (**C**) GR, (**D**) KK, (**E**) RR, (**F**) RK, and (**G**) KR; the pink square represents the basic polar residues, the blue square represents the acid polar residues, the green square represents the uncharged polar residues, and the yellow square represents the nonpolar residues. (**H**) The secondary structure prediction of FA, GK, GR, KK, RR, RK, and KR. The CD spectra of (**I**) FA, (**J**) GK, (**K**) GR, (**L**) KK, (**M**) RR, (**N**) RK, and (**O**) KR; the solvents were dissolved in 10 mM PBS (pH 7.4, yellow line), 50% TFE (pink line), or 30 mM SDS (blue line); the illustrations were three-dimensional structure modeling of the SAMPs.

The sequences and pertinent property parameters of the newly designed SAMPs are detailed in Table 1. The purity, as confirmed by HPLC analyses, of the synthesized SAMPs exceeded 95%, and the actual molecular weights (Table 1; Fig. S1) obtained through mass spectrometry (MS) closely matched their theoretical molecular weights, indicating the successful synthesis of the SAMPs. As listed in Table 1, all six designed SAMPs were positively charged, with net charges ranging from +5 to +6 (GK, GR: +5; KK, RR, RK, KR: +6), surpassing the natural peptide FA with a net charge of +2. Meanwhile, the six designed SAMPs demonstrated a consistent hydrophobicity rate of 60% (Table 1), which was lower than FA (73%). Given that the hydrophobic amino acid residues of most effective AMPs typically constitute 40%–60% of the total amino acid residues (18, 21, 25), the reduced hydrophobic proportion in the designed SAMPs is noteworthy. Furthermore, as indicated by the online helical wheel prediction (Fig. 3A through G), SAMPs exhibited amphiphilic helical wheel configurations characterized by partially interrupted hydrophobic and cationic surfaces. Notably, compared with FA, GK, and GR, the distribution of hydrophilic (pink, blue, and green) and hydrophobic (yellow) amino acid residues in the remaining four SAMPs displayed a more uniform pattern on both sides of the helical wheel, resulting in a more symmetrical structure. Previous studies have proven that the symmetrical α-helix structure is beneficial to improve antibacterial efficacy and mitigate mammalian cytotoxicity (26, 27). The secondary structure prediction of SAMPs shows that they have the potential to replicate the α-helical structure found in the parent peptide FA, whereas GK and GR display a slightly truncated helical structure (Fig. 3H), possibly due to alterations in the symmetrical amphiphilic structure resulting from the insertion of G. In addition, the actual secondary structure of the SAMPs was analyzed using circular dichroism (CD) in various solvents, including 10 mM PBS (pH 7.4, mimicking the aqueous environment), 50% 2,2,2-trifluoroethanol (TFE; mimicking the hydrophobic environment of the microbial membrane), and 30 mM SDS micelles (mimicking the negatively charged environment of prokaryotic membrane). As shown in Fig. 3I through O, all the designed SAMPs displayed minimal order in 10 mM PBS. Specifically, the KK, RR, RK, and KR spectra exhibited a weak negative peak around 208 nm, indicating the potential for helical conformation formation. In the presence of 50% TFE, all SAMPs exhibited a tendency toward folding a typical α-helical conformation, as evidenced by the characteristic double minima at 208 nm and 222 nm. Although the α-helical tendency was still observed in 30 mM SDS micelles, it was not as strong or pronounced as in TFE, evident from the reduced negative peak at 222 nm. Simultaneously, it was observed that RK and KR exhibited a pronounced inclination toward forming an α-helical conformation, aligning with the outcomes derived from three-dimensional (3D) structural modeling (illustrations in Fig. 3I through O). The interplay of hydrophobicity, charge density, amphiphilic nature, and additional structural attributes is anticipated to significantly influence the antibacterial efficacy of SAMPs.

### *In vitro* antimicrobial activity and stability of SAMPs

**Fig 4:**
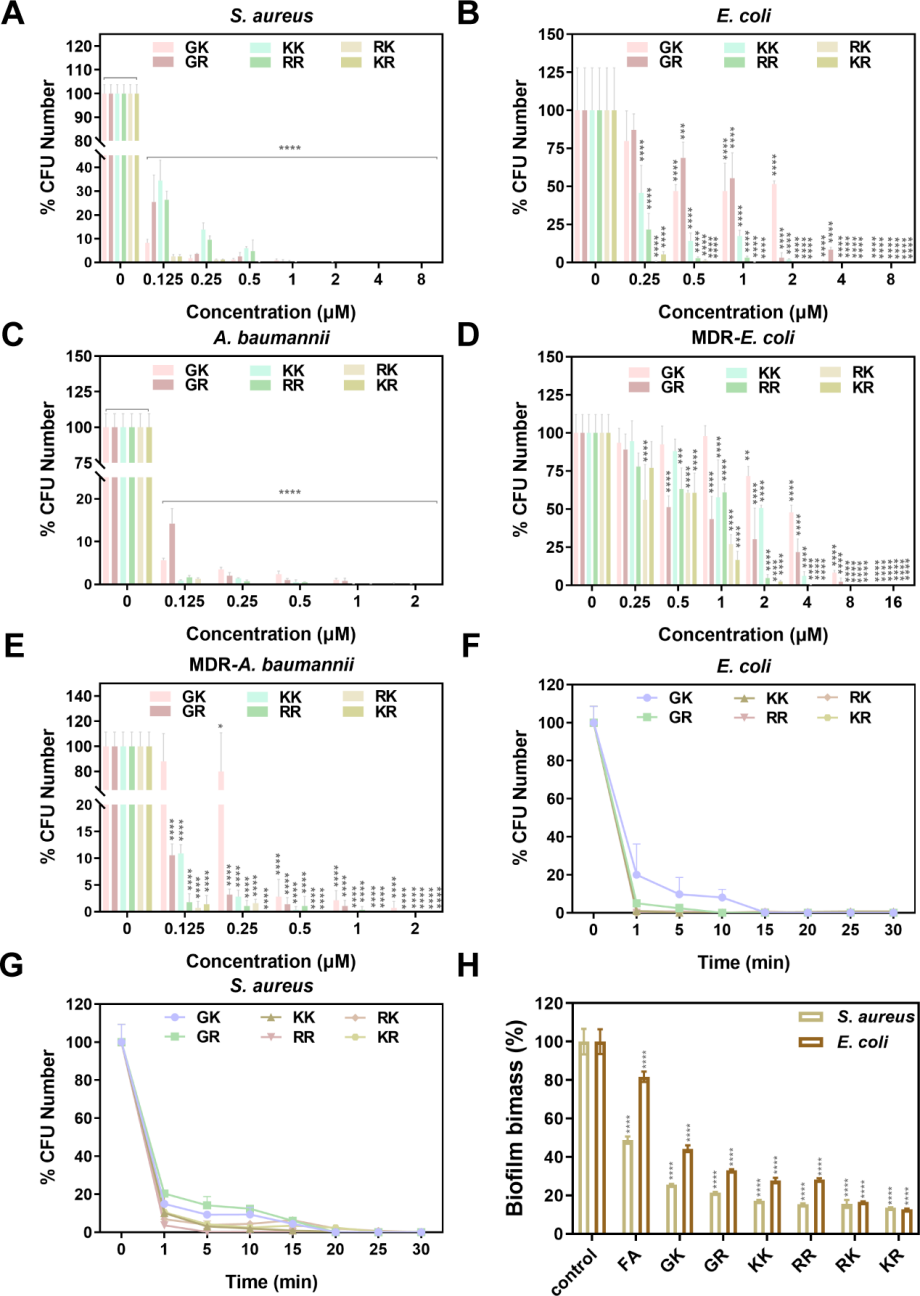
Antimicrobial and antibiofilm activity of SAMPs. Survival colony counts of (**A**) *S. aureus*, (**B**) *E. coli*, (**C**) *A. baumannii*, (**D**) MDR-*E. coli,* and (**E**) MDR-*A. baumannii* treated with SAMPs (0.125–16 μM). Kill-time kinetics of SAMPs (1 × MBC) against (**F**) *E. coli* and (**G**) *S. aureus*. (**H**) Biofilm quantification of *S. aureus* and *E. coli* stained by crystal violet after treatment with SAMPs (8 μM). Data are presented as mean ± SD (*n* = 3); *P < 0.05, **P < 0.01, ***P < 0.001, ****P < 0.005, and ns means no significant difference.

The minimal inhibitory concentrations (MICs) and minimum bactericidal concentrations (MBCs) against a panel of clinically relevant Gram-positive bacteria, *Staphylococcus aureus* (*S. aureus*) and Gram-negative bacteria, *E. coli*, *Acinetobacter baumannii* (*A. baumannii*), MDR-*E. coli*, and MDR-*A. baumannii,* were determined and summarized in Table 2 to evaluate the antibacterial effectiveness of SAMPs. Initial analysis of Table 2 and Fig. 4A through C, focusing on the three prevalent pathogenic bacteria (*S. aureus*, *E. coli,* and *A. baumannii*), indicated that six specific SAMPs exhibited enhanced activity (0.25 µM < MIC < 2 µM, 0.25 µM < MBC < 8 µM) than FA (MIC >32 µM, MBC >128 µM). Subsequent research involving MDR bacteria demonstrated the efficacy of synthetic SAMPs against MDR-*E. coli* and MDR-*A. baumannii*, achieving MBC values at the nanomolar (nM) level (Table 2; Fig. 4D and E). The antimicrobial potency of the engineered SAMPs surpassed that of FA by over a hundredfold, a phenomenon attributed to the high membrane interface affinity of W and L, as well as the positive charge carried by K and R, facilitating strong binding of the SAMPs to the negatively charged phospholipid layer of the membrane. Among them, KR and RK emerged as the most potent antimicrobials exhibiting MICs ranging from 0.25 to 2 µM against both common pathogenic bacteria and MDR bacteria. Meanwhile, KR and RK were able to effectively eradicate over 99.9% of bacteria at concentrations as low as 0.5–4 µM (Fig. 4A through E). In contrast, the antibacterial efficacy of KK and RR, which share similar positive charge and hydrophobicity characteristics with KR and RK (Table 1), was slightly inferior. This difference may be attributed to the more pronounced α-helical structure folding observed in KR and RK (Fig. 4N and O), suggesting that the K-R pairs play a more prominent role in promoting the antibacterial activity. Despite both of the SAMPs displaying interrupted cationic surfaces, their helical structures were relatively symmetric and capable of forming α-helical conformations. Coupled with the ideal net charges and appropriate hydrophobic rates, KR and RK demonstrated superior antimicrobial activity. This observation is corroborated by the fact that α-helix formation is a major driver of peptide insertion into the lipid bilayer (28, 29). In contrast, the less effective SAMPs GK and GR (0.5 µM < MIC < 16 µM, 1 µM < MBC < 16 µM) exhibited the lowest positive charge among the peptides analyzed (Table 1). This study provides evidence that the presence of doubly positively charged amino acids, such as K and R, could enhance the antibacterial activity by potentially inducing steric hindrance and charge repulsion (30). It is widely acknowledged that AMPs bind to bacterial anionic membranes through electrostatic interactions, in which cations are the key prerequisites (31). The comparison of six designed SAMPs with the natural peptide FA reveals differences in charge distribution, hydrophobicity, and amphiphilic structure, resulting in varied antibacterial efficacy. These findings confirm that the amino acid composition accounts for the major contribution to the antimicrobial spectrum, encompassing factors such as net charge, hydrophobicity, amphipathicity, and other parameters (32). In contrast, the MIC results of Colistin E (Table S1) showed that Colistin E only exhibited varying degrees of antibacterial activity against negative bacteria such as *E. coli*, *A. baumannii*, and MDR-*A. baumannii*. However, no significant antibacterial activity was detected against MDR-*E. coli*, and the MIC values were all above 32 µM, significantly higher than the MICs of SAMPs. At the same time, the MIC values of common antibiotics GSS for MDR bacteria were all above 256 µM (Table S1). The antibacterial activity of the designed SAMPs was more than 8–100 times higher than that of these representative antibiotics. Overall, the MIC and MBC values of SAMPs against various bacterial strains were within a similar order of magnitude, implying that these designed SAMPs have the potential to display broad-spectrum antimicrobial properties at lower concentrations against both Gram-negative, Gram-positive, and MDR bacteria.

**TABLE 2:** The MICs and MBCs (μM) of SAMPs

Name	*S. aureus*		*E. coli*		*A. baumannii*		MDR-*E. coli*		MDR-*A. baumannii*	
	MIC	MBC	MIC	MBC	MIC	MBC	MIC	MBC	MIC	MBC
FA	>32	>128	>32	>128	>32	>128	>32	>128	>32	>128
GK	1	2	2	8	2	4	16	16	1	4
GR	1	2	2	8	2	2	16	16	0.5	2
KK	0.5	2	0.5	4	0.5	1	2	8	0.5	1
RR	0.5	2	0.5	2	1	1	2	8	0.5	2
RK	0.25	1	0.25	1	0.5	0.5	2	4	0.25	0.5
KR	0.25	0.5	0.25	0.5	0.25	0.25	1	4	0.25	0.25

Notably, six engineered SAMPs showed extremely quick killing kinetics. Specifically, KR, RK, KK, and RR at 1× MBC resulted in almost complete eradication of *E. coli* cells within 1 min, while GK and GR achieved 99% cell death within 10–15 min (Fig. 4F). In the case of *S. aureus*, KR, RK, KK, and RR (1× MBC) were able to eliminate more than 90% of cells within 5 min, with complete eradication by all six SAMPs within 20 min (Fig. 4G). These findings demonstrate the rapid bactericidal efficacy of our designed SAMPs, with the slightly slower killing kinetics of GK and GR, potentially attributed to their relatively lower charge number and shorter helical structure.

In a multifaceted physiological milieu, the limited stability of peptide-based drugs severely constrains their potential clinical applications (12). Given that salts, serum, elevated temperatures, and acid-base can hinder the antimicrobial activity of peptides, we conducted an assessment of the physiological stability of designed SAMPs, despite their demonstrated efficacy and rapid bactericidal action. Initially, we subjected the SAMPs to extreme conditions of high temperature (60°C, 80℃) and acid-base (pH 5.5, 8.5) and observed minimal impact on their antibacterial properties (Table 3). Previous reports have indicated that the charge-shielding effect of cationic salts can diminish or abolish the activity of peptides (20, 33, 34). Specifically, monovalent or multivalent free cations (e.g., Na^+^ and K^+^) can competitively attach to anionic groups on bacterial membranes, weakening the electrostatic interaction between AMPs and bacterial membranes, as well as enhancing membrane rigidity, ultimately diminishing the efficacy of AMPs (35–38). Fortunately, the SAMPs have demonstrated resistance to inactivation by monovalent salt cations. The MICs of SAMPs remained unchanged following treatment with 4.5 mM KCl, while the presence of Na^+^ (150 mM NaCl) resulted in a slight reduction in the antibacterial activity of KK and RR by a factor of 2 (Table 3). This observation may be attributed to the higher abundance of cationic amino acid pairs of K-R in KR and RK, which contribute to the formation of a stable helical structure that enhances resistance to salt (39, 40) and facilitates the electrostatic adsorption between the SAMPs and the anionic microbial membranes (20). Following this, we conducted an examination of the serum stability of SAMPs. Serum has been shown to detract from the antimicrobial activity of AMPs primarily through serum protease degradation and the presence of anionic proteins, resulting in a reduced half-life of only a few minutes (41, 42). Despite exposure to serum for a period of 12 h, the MICs of the SAMPs remained consistent (Table 3), implying that serum has a minimal impact on the antimicrobial properties of the SAMPs. The aforementioned findings suggest that our AMPs design strategy successfully enhanced the stability of peptides against diverse physiological conditions, which is also related to the swift bactericidal rate of SAMPs: bacteria are eradicated within a brief timeframe, insufficient for the degradation of peptides.

**TABLE 3:** The MICs (μM) of SAMPs after different treatments (salt, serum, thermal, and acid−base)

Name	*S. aureus*							*E. coli*						
	60°C	80°C	pH 5.5	pH 8.5	NaCl	KCl	Serum	60 ℃	80°C	pH 5.5	pH 8.5	NaCl	KCl	Serum
GK	1	1	1	1	1	1	1	2	2	2	2	2	2	2
GR	1	1	1	1	1	1	1	2	2	2	2	2	2	2
KK	0.5	0.5	0.5	0.5	1	0.5	0.5	0.5	0.5	0.5	0.5	1	0.5	0.5
RR	0.5	0.5	0.5	0.5	0.5	0.5	0.5	0.5	0.5	0.5	0.5	1	0.5	0.5
RK	0.25	0.25	0.25	0.25	0.25	0.25	0.25	0.25	0.25	0.25	0.25	0.25	0.25	0.25
KR	0.25	0.25	0.25	0.25	0.25	0.25	0.25	0.25	0.25	0.25	0.25	0.25	0.25	0.25

### *In vitro* antibiofilm activity of SAMPs

**Fig 5:**
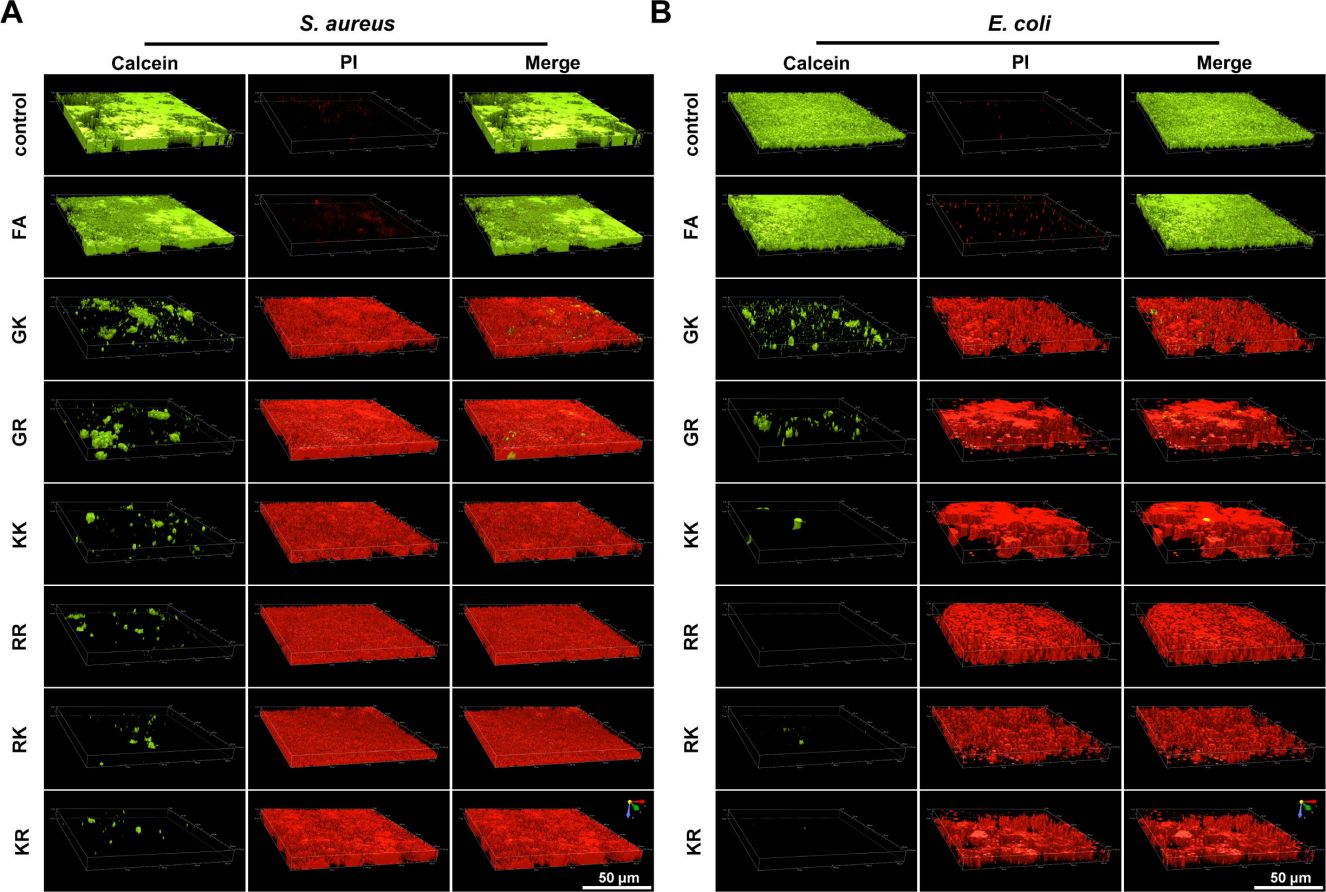
Antibiofilm activity of SAMPs. The 3D CLSM images of Calcein-AM/PI-stained (**A**) *S. aureus* and (**B**) *E. coli* biofilms treated with SAMPs (8 μM). (Red: PI, excitation wavelength is 535 nm, emission wavelength is 615 nm; Green: Calcein, excitation wavelength 494 nm, emission wavelength 514 nm). Scale bar: 50 µm.

Biofilm formation serves as a mechanism for bacteria to adapt and develop resistance to antimicrobial agents, contributing to the burgeoning AMR crisis (37). The limited permeability of antibiotics through the extracellular polymeric substance matrix leads to the intrinsic resistance of bacterial communities to antibiotics (43). In light of AMPs being considered as potent agents against MDR bacteria and biofilms (44), we conducted further experiments to assess the effectiveness of synthetic SAMPs in inhibiting biofilm formation by impeding bacterial growth in the early stages and eradicating the formed mature biofilms (45). The results demonstrate that the designed SAMPs significantly suppressed the initial proliferation of biofilm formed by both *S. aureus* and *E. coli*. Figure S2 displays images of the bacterial biofilms stained with crystal violet following treatment with various SAMPs. Treatment with six SAMPs led to a notable decrease in crystal violet-stained cells, disruption of biofilm formation, and a significant decrease in membrane density compared to both the control group and the native peptide FA group. Additionally, the biomass of the remaining biofilms was also quantified in Fig. 4H. The study revealed a notable decrease in the intracellular crystal violet content of bacteria representing the biofilm mass to below 20% following KR and RK treatment, aligning with the antibacterial effect against planktonic bacteria. The AMPs’ ability to prevent biofilm formation is partially due to their direct bactericidal effects on planktonic bacteria (28). Additionally, inhibiting the adhesion of planktonic bacteria to surfaces also plays a role in preventing biofilm formation (46). The challenge of eradicating mature biofilms presents a significant clinical obstacle, prompting further investigation into the potential of AMPs to disrupt preformed biofilms. Following a 7-day incubation period, the formed biofilms of *S. aureus* and *E. coli* were treated with different SAMPs and evaluated using 3D confocal laser scanning microscopy (CLSM). It is evident from Fig. 5A and B that a significant quantity of live cells, indicated by green fluorescence, was present in both the untreated control group and the native peptide FA group. Conversely, a substantial amount of red fluorescence, signifying dead bacteria, was observed when the biofilm was subjected to six SAMPs, particularly KK, RR, KR, and RK, indicating the efficacy of these SAMPs in eradicating bacteria within the established biofilm. In a nutshell, these newly developed SAMPs presented here are equipped with the potential utility in addressing the mounting challenge posed by biofilms and MDR microorganisms.

### Preliminary mechanistic studies

**Fig 6:**
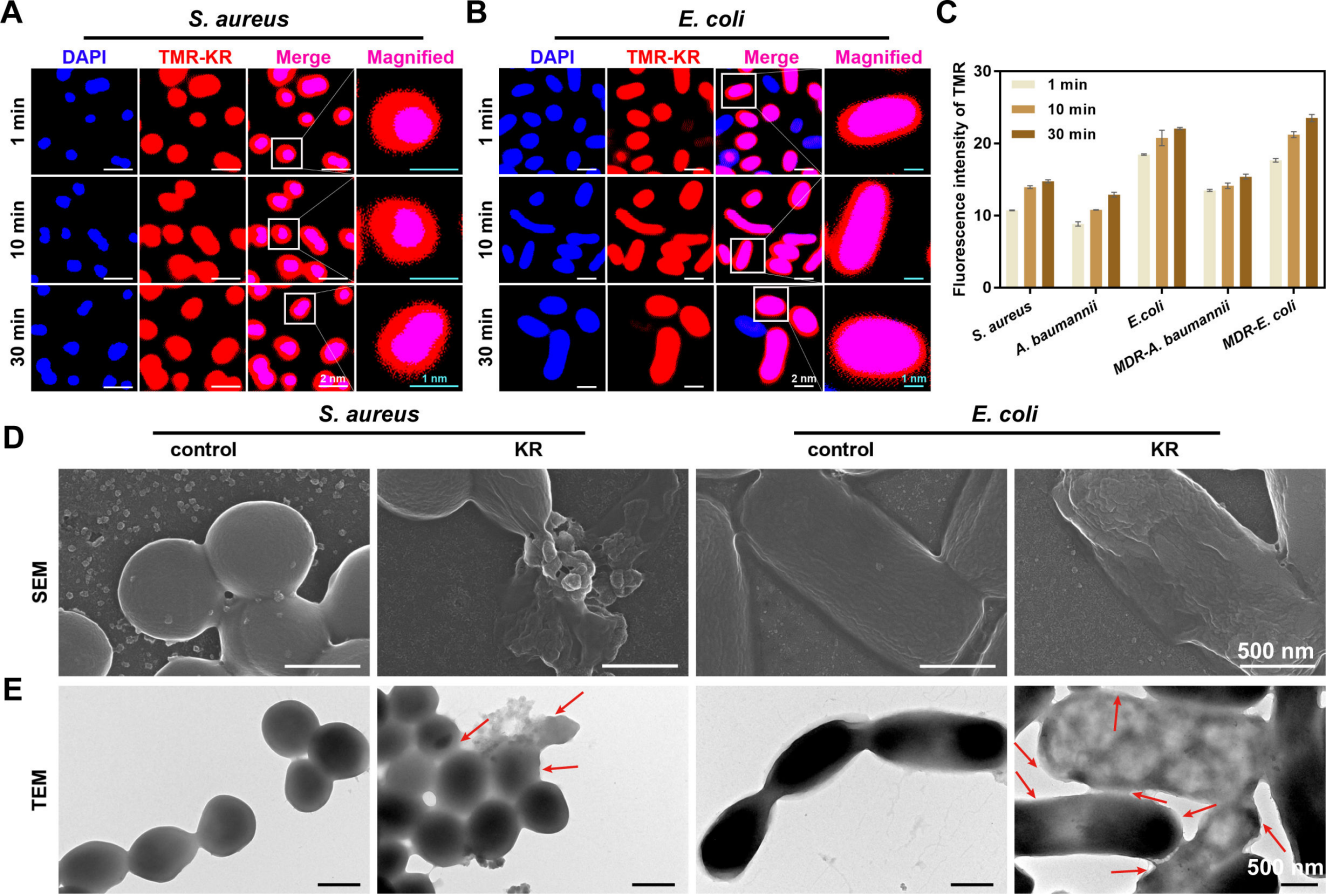
Membrane localization and damage of SAMPs. Confocal laser microscope photos of DAPI-stained (**A**) *S. aureus* and (**B**) *E. coli* treated with TMR-KR (0.2 μM) for 1, 10, and 30 min, and their (**C**) fluorescence intensity quantification. (Red: TMR-KR, excitation at 558 nm, emission at 586 nm; Blue: DAPI, excitation at 340 nm, and emission at 488 nm; Pink: Merge, the overlap of red fluorescence and blue fluorescence). White scale bar: 2 µm, green scale bar: 1 µm. (**D**) SEM and (**E**) TEM photos of *S. aureus* and *E. coli* treated with KR (2 μM). Scale bar: 500 nm. Regions of disruption are indicated by red arrows.

Models of AMP-induced transmembrane perturbations, such as barrel walls, carpets, electroporation, depolarization, and toroidal pores (38), have been proposed as typical action mechanisms of AMPs. Due to its superior performance in antimicrobial activity, antibiofilm activity, and stability, KR was chosen for further investigation into its antibacterial mechanism. To investigate the interaction between SAMPs and bacterial cells, 5-carboxyl tetramethyl-rhodamine (TMR) fluorescein was used to label KR for precise intracellular localization. TMR fluorescein modified KR (TMR-KR) was synthesized successfully (Fig. S1H) and then incubated with bacteria stained with DAPI for varying durations (1 min, 10 min, and 30 min) to capture intracellular tracer fluorescence images. Analysis of the obtained images (Fig. 6A and B; Fig. S3A through C) revealed uniform concentration of TMR-KR on the membrane surfaces and rapid internalization by cells within 1 min, as indicated by the presence of red signal on the cell surface and the merged pink signal of TMR-KR (red) and DAPI (blue). The rapid translocation of SAMPs across the cell membrane within 1 min, as evidenced by bactericidal kinetics, is a key factor contributing to their fast bactericidal efficacy. Simultaneously, the increase in intracellular fluorescence intensity of TMR-KR over time (Fig. 6C) suggests a gradual internalization of SAMPs across the cell membranes, ultimately leading to accumulation on DNA. These findings indicate that SAMPs may target both the bacterial membrane and intracellular components.

Aiming at observing the membrane action mechanism more directly and visually, the morphological changes in *S. aureus* and *E. coli* induced by KR were further examined using scanning electron microscopy (SEM) and transmission electron microscopy (TEM). The SEM micrographs in Fig. 6D clearly depict the smooth and intact membranes of untreated bacterial cells. Conversely, following treatment with KR, the surface of bacterial cells exhibited signs of damage, including breakage, roughness, wrinkling, atrophy, large holes, and isolated fragments. Additionally, the TEM images (Fig. 6E) also revealed that bacterial cells in the control groups exhibited intact membrane structure. After treatment with KR, the cell membranes were significantly damaged, along with pores that traversed the membrane structure, cell fragments, and even leakage of the intracellular contents. It is our contention that the extensive physical disruption of the membranes caused by the SAMPs is the primary factor contributing to bacterial death.

**Fig 7:**
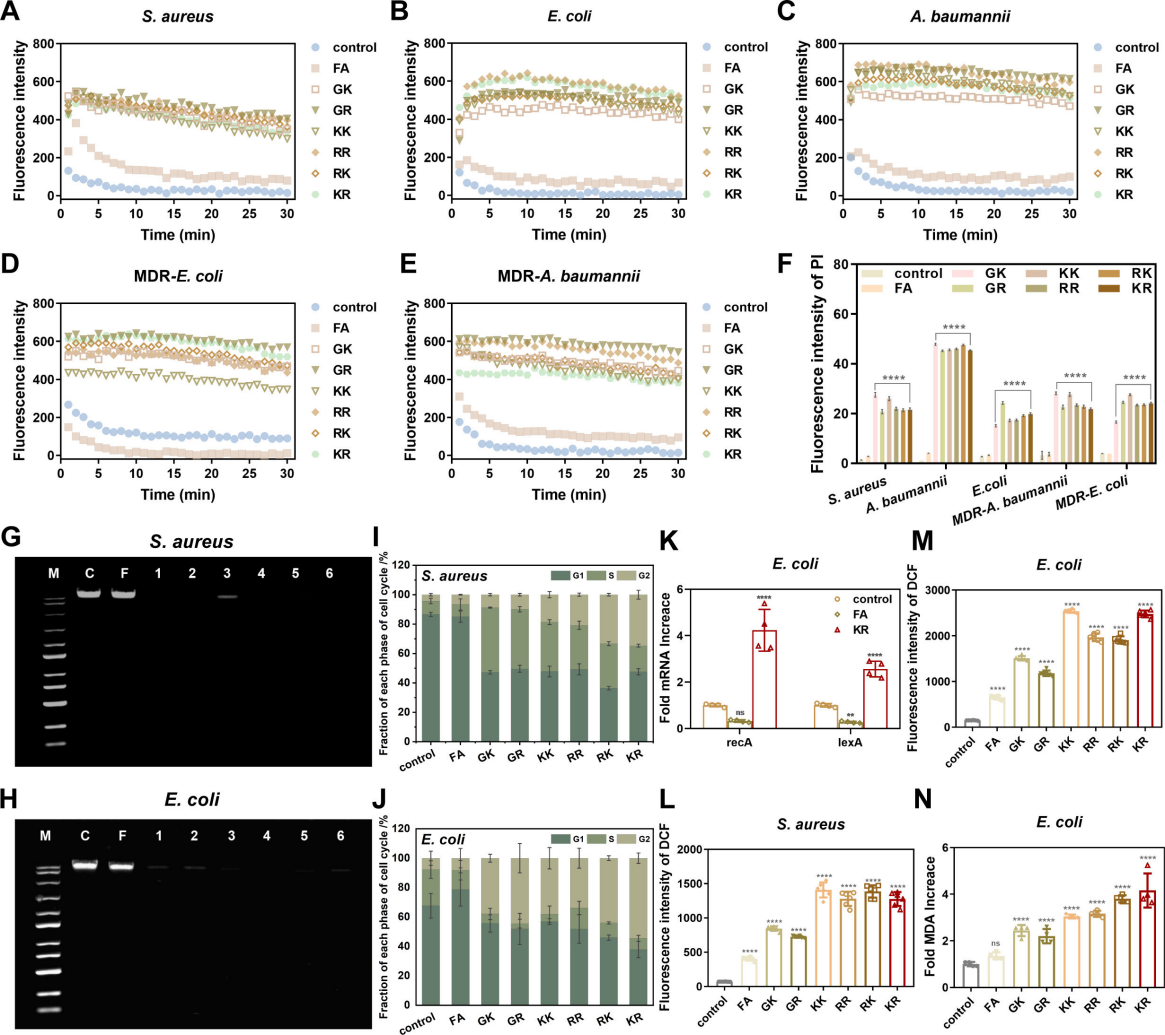
Action mechanism of SAMPs. CM depolarization of (**A**) *S. aureus*, (**B**) *E. coli*, (**C**) *A. baumannii*, (**D**) MDR-*E. coli,* and (**E**) MDR-*A. baumannii* induced by SAMPs (2 μM); excitation wavelength 622 nm, emission wavelength 670 nm. (**F**) Membrane permeability of *S. aureus*, *E. coli*, *A. baumannii*, MDR-*E. coli,* and MDR-*A. baumannii* induced by SAMPs (0.2 μM); excitation wavelength 535 nm, emission wavelength 615 nm. Genomic DNA binding affinity detection of SAMPs (2 μM) with (**G**) *S. aureus* and (**H**) *E. coli*. M, DNA Maker; C, blank control; F, FA; 1, GK; 2, GR; 3, KK; 4, RR; 5, RK; 6, KR. Cell cycle detection of (**I**) *S. aureus* and (**J**) *E. coli* treated with SAMPs (0.2 μM). (**K**) SOS-related gene expression level of *E. coli* induced by SAMPs (0.2 μM). Fluorescence intensity quantification of (**L**) *S. aureus* and (**M**) *E. coli* staining by DCFH-DA after treatment with SAMPs (0.2 μM). (**N**) MDA content detection of *E. coli* after treatment with SAMPs (0.2 μM). Data are presented as mean ± SD (*n* = 3); **P < 0.01, ****P < 0.005, and ns means no significant difference.

Based on these findings, we posited that SAMPs primarily localize on the bacterial membrane, resulting in membrane disturbance. Additionally, they may inactivate bacteria by transferring into the cells and binding to intracellular targets, such as DNA. To test this hypothesis, we first examined changes in the cytoplasmic membrane (CM) potential of bacteria induced by the SAMPs. Figure 7A through E illustrates a significant increase in relative fluorescence intensity after treatment with SAMPs compared to the control and FA. Overall, the SAMPs exhibited greater CM depolarization capabilities against the tested bacteria compared to FA were able to induce rapid changes in membrane potential within 1 min, which correlated with the kill rate of SAMPs. This result implies that the unregulated ion flux and subsequent membrane potential dissipation destabilization resulting from membrane perturbations may serve as a major mechanism for membrane disruption (3). Meanwhile, propidium iodide (PI) was used to assess the integrity of the bacterial membrane. According to the flow cytometry data presented in Fig. 7F and Fig. S3D, an increased PI signal was observed in the SAMP-treated groups, indicating heightened membrane permeability and compromised membrane integrity after treatment with SAMPs. Notably, this robust and swift membrane disruption response aligns with the rapid bactericidal rate of SAMPs, supporting the notion that cell death was initiated by escalating CM depolarization and permeability, ultimately resulting in the release of cellular contents and physical integrity loss as evidenced by SEM and TEM.

In addition to the typical membrane lysis, the intracellular mechanisms of SAMPs were initially investigated through a DNA-binding test. The diminished bands observed in the bacterial genome DNA of the six groups treated with SAMPs indicated a significant binding of these molecules to nucleic acids upon cellular entry (Fig. 7G and H; Fig. SE through G), suggesting intracellular effects may act as an additional bactericidal mechanism. To further elucidate the intracellular physiological effects related to DNA induced by SAMPs, the cell cycle was subsequently analyzed using flow cytometry. Figure 7I and J demonstrates a notable shift in the allocation ratio of cell cycle phases following treatment with SAMPs. Specifically, there was a significant increase in the S and G2 phases of both *S. aureus* and *E. coli* cells, accompanied by a decrease in the G1 phase, compared to the control and FA groups. This phenomenon indicated that the designed SAMPs could interfere with the DNA replication and induce DNA damage by arresting bacterial cell cycles in the S and G2 phases, thereby inhibiting crucial cellular physiological processes.

Prior research has demonstrated that cellular stress, such as membrane depolarization and severe DNA damage verified above, could activate bacteria’s programmed cell death (PCD) pathways (47, 48). The SOS response system, related to recA and lexA genes (47, 48), is related to the induction of cell cycle arrest and apoptosis. Based on gene expression studies, KR at a sub-lethal level could induce an increase in recA and lexA by more than four times and two times, respectively, while FA induced almost no change (Fig. 7K), suggesting that KR may induce SOS pathways in *E. coli*. Meanwhile, we also detected FITC-labeled Annexin-V fluorescence signals in KR-treated *E. coli* (Fig. S3H), further demonstrating the triggering of the apoptosis program (49). Moreover, we quantified the levels of intracellular reactive oxygen species (ROS) following KR treatment (Fig. 7L and M; Fig. S3I through K), which have been considered as potent inducers of SOS pathways (49, 50). Obviously, SAMPs triggered the excessive production of ROS in bacteria and led to an increase in malondialdehyde (MDA) levels, which is a product of membrane lipid peroxidation (Fig. 7N). Taken together, these findings support our earlier hypothesis regarding the multiple antimicrobial mechanisms of SAMPs: the action of SAMPs results in membrane perturbation, rapid accumulation of ROS and lipid oxidation in bacterial membranes; simultaneously, SAMPs are internalized to bind to bacterial DNA, interfering with the normal cell cycle program and activating apoptotic pathway; ultimately, the synergistic effects of membrane damage, DNA damage, and oxidative stress result in bacterial death and cell lysis.

**Fig 8:**
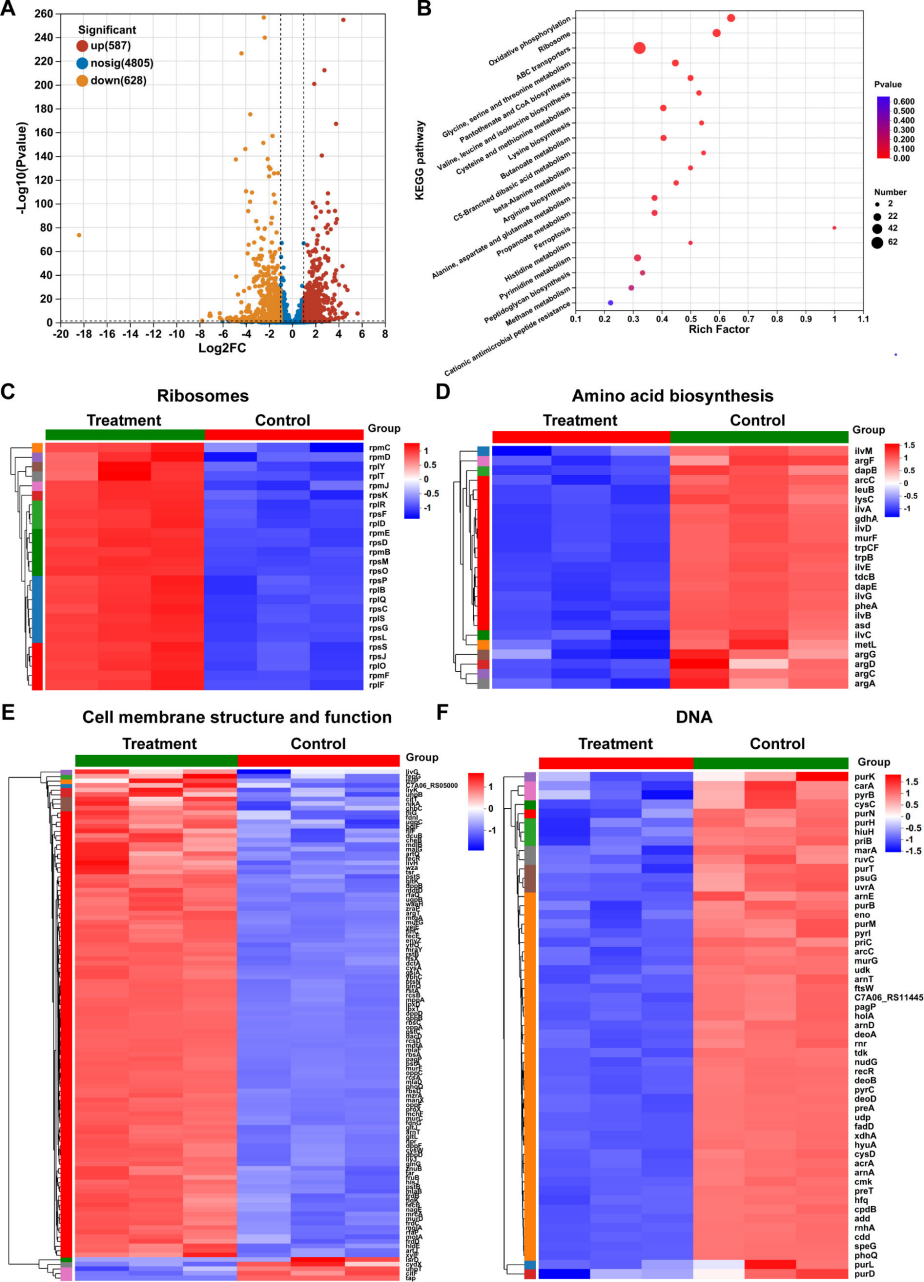
The transcriptome analysis of *E. coli* after treatment with KR (0.2 μM, *n* = 3). (**A**) The volcanic map of DEGs; the horizontal dotted line is −log_10_(*P*) = −log_10_(0.05), and the vertical virtual line is |log_2_(fold change)| = 1. (**B**) The KEGG enrichment analysis of DEGs (*P* values < 0.05). The number of genes enriched in this pathway was represented by the size of the bubble, and the enriched *P* value was represented by the color of the bubble. Heat map of (**C**) ribosome-related, (**D**) amino acid biosynthesis-related, (**E**) cell membrane structure and function-related, and (**F**) DNA-related differential gene expression. (*P* values < 0.05 and |log_2_(fold change)| > 1); blue indicated downregulated genes, while red indicated upregulated genes.

The intracellular mechanisms of AMPs are still under investigation (51), with studies focusing on targets (15) of cell wall biosynthesis (52), ribosome translation (53), energy metabolism (54), and so on. Inspired by previous research, we utilized the transcriptome sequencing analysis to examine the transcriptional changes of genes in *E. coli* following treatment with KR. Our findings revealed a total of 6,020 genes identified, with 1,215 significantly differentially expressed genes (DEGs) in *E. coli* after KR treatment, including 587 upregulated genes and 628 downregulated genes (Fig. 8A). Furthermore, our analysis of Fig. 8B demonstrated enrichment of 20 pathways in the Kyoto Encyclopedia of Genes and Genomes (KEGG) database, indicating the most significant associations of the aforementioned significant DEGs with the ribosome, amino acid biosynthesis, ABC transporters, metabolism processes, etc. The data serve as a reminder that KR does have the potential to elicit extensive alterations in bacterial physiological functions by targeting various components such as biosynthesis, metabolism, and membrane proteins. In line with our previous conjecture, these designed SAMPs exhibit distinct mechanisms of action compared to conventional antibiotics with narrow target specificity (55), potentially reducing the likelihood of resistance development.

The ribosome, composed of RNA and protein, functions as the central hub for protein synthesis in biological systems (56). The small ribosomal 30S subunit, responsible for decoding mRNA, and the large ribosomal 50S subunit, which catalyzes peptidyl transfer (56, 57), are crucial components in the ribosome assembly and protein biosynthesis. Normal expression of these subunits may be a decisive factor in the survival of bacterial cells (58). According to KEGG enrichment analyses, there were 26 significantly upregulated (2.057- to 4.592-fold) and 2 downregulated (0.35- to 0.376-fold) genes associated with 30S and 50S subunit ribosomal proteins in *E. coli* after KR treatment (Fig. 8C and 4), indicating that KR may interfere with the normal synthesis of ribosomal protein subunits. The initiation of protein synthesis requires the 30S and 50S ribosomal protein subunits (59), and any abnormalities in these ribosomal protein subunits induced by KR may disrupt intracellular protein synthesis. Analysis presented in Fig. 8D and Fig. S5 and S6 indicates that the majority of the genes encoding lysine biosynthesis and arginine biosynthesis exhibited downregulation ranging from 0.272- to 0.459-fold, with the exception of the murF, dapE, and gdhA genes, which showed upregulation by 2.488- to 4.19-fold. Moreover, the genes involved in valine, leucine, and isoleucine biosynthesis and phenylalanine, tyrosine and tryptophan biosynthesis pathway (except for the ilvC gene, which was upregulated of 0.47-fold) were significantly upregulated by 2.149- to 11.114-fold (Fig. 8D; Fig. S7 and S8), which means that KR may potentially disrupt amino acid and protein synthesis by interfering with ribosome biogenesis.

Meanwhile, the differential expression of genes related to cell membrane structure and function was also observed in *E. coli* after treatment with KR (Fig. 8E), including 53 ABC transporter genes (Fig. S9), 37 two-component system genes (Fig. S10), 7 phosphotransferase system (Fig. S11), 8 peptidoglycan biosynthesis-associated genes (Fig. S12), 8 lipopolysaccharide biosynthesis-associated genes (Fig. S13), and 4 flagellar assembly genes (Fig. S14). It can be inferred from this result that KR may impair the composition and function of cell membranes and walls, leading to a decrease in bacterial migration led by flagella.

Furthermore, the expression of cellular carbohydrate metabolism and energy metabolism-related genes in *E. coli* showed notable variations following treatment with KR (Fig. S15). These differences encompassed oxidative phosphorylation genes (Fig. S16), glycine, serine, and threonine metabolism genes (Fig. S17), glycolysis/gluconeogenesis genes (Fig. S18), pentose phosphate pathway genes (Fig. S19), sulfur metabolism pathway genes (Fig. S20), methane metabolism genes (Fig. S21), pyruvate metabolism pathway genes (Fig. S22), C5-branched dibasic acid metabolism genes (Fig. S23), etc. This indicates that the administration of KR may induce significant alterations in energy generation and other metabolic pathways in bacteria.

In addition, many DNA-related genes were differentially expressed in *E. coli* after treatment with KR (Fig. 8F), which are involved in purine and pyrimidine metabolism (Fig. S24 and S25), homologous recombination (Fig. S26), cell cycle (Fig. S27), ferroptosis (Fig. S28), etc. It can be seen that KR may further impact the function of the intracellular targets beyond its rapid membrane-disrupting effects.

To sum up, it can be tentatively concluded that the antimicrobial efficacy of SAMPs is contingent upon a comprehensive multimodal mechanism that extends beyond mere membrane damage, encompassing the modulation of various physiological indicators, such as cell membrane functionality, transcription, translation, metabolic processes, and so on. Nevertheless, further investigation is still warranted to ascertain the precise interaction of SAMPs with intracellular targets.

### *In vivo* biocompatibility evaluation of SAMPs

**Fig 9:**
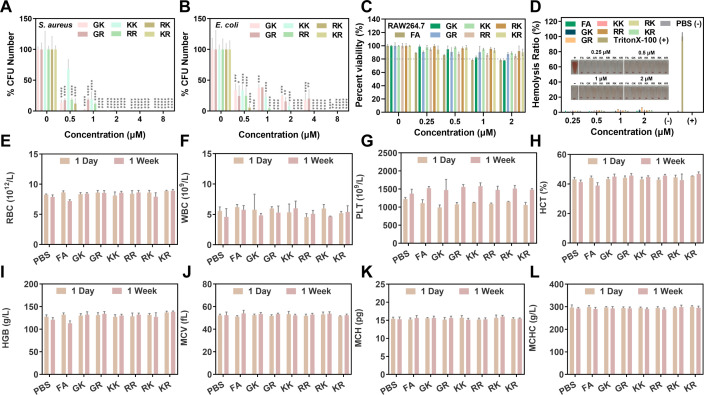
The biocompatibility analysis of SAMPs. Survival colony counts of (**A**) *S. aureus* and (**B**) *E. coli* after drug resistance induction with SAMPs (0.5× MIC). (**C**) Survival rate of RAW264.7 cells treated with FA and six designed SAMPs (0–2 μM). (**D**) Hemolytic assays of FA and six designed SAMPs (0–2 μM); Triton X-100 as positive (+) control, PBS as negative (−) control. Hematological parameters of (**E**) RBC, (**F**) WBC, (**G**) PLT, (**H**) HCT, (**I**) HGB, (**J**) MCV, (**K**) MCH, and (**L**) MCHC in mice on days 1 and 10 after tail vein injection of FA and six designed SAMPs (5 mg kg^−1^). Data are presented as mean ± SD (*n* = 3); *P < 0.05, **P < 0.01, ***P < 0.001, ****P < 0.005.

Prior to conducting efficacy studies at the *in vivo* level, we evaluated the biocompatibility of SAMPs due to the historical concerns regarding their safety, particularly mammalian cytotoxicity. The initial evaluation focused on the development of resistance to the SAMPs, with Colistin E serving as a control. The results depicted in Fig. S29 indicate a significant decrease in the antimicrobial activity of Colistin E following the assay period. Conversely, the MBCs of the SAMPs against *S. aureus* and *E. coli* remained consistent after continuous passage at sub-MIC concentrations (Fig. 9A and B), meaning a lack of spontaneous resistance development to the SAMPs was observed. This phenomenon can be attributed to the multimodal antimicrobial mechanism of SAMPs, which hinders bacteria from altering their membrane structure, intracellular targets, and other factors to resist injury (14, 60). Subsequently, RAW264.7 cells were selected as the subject of investigation to assess the mammalian cytotoxicity of the SAMPs. Six SAMPs showed negligible cytotoxicity, maintaining cell viability at approximately 80%–90% within the effective bactericidal concentration range *in vitro* (Fig. 9C). This may be attributed to the formation of a symmetric amphiphilic structure, weakened hydrophobic interactions, and reduced affinity with the neutral mammalian cell membrane. Meanwhile, the hemolytic activity of six SAMPs was then evaluated and depicted in Fig. 9D, revealing weak hemolytic activity below 6% hemolysis at effective bactericidal concentrations. These SAMPs demonstrated reliable *in vitro* biocompatibility by selective targeting of bacterial cells over mammalian cells, which is mainly due to the contrasting cell membrane charges of eukaryotic (electrically neutral) and prokaryotic cells (negative) (61). This disparity in membrane potential renders cationic AMPs more inclined to bind to negatively charged bacterial cell membranes, thereby minimizing harm to mammalian cells. To conduct *in vivo* trials, we injected SAMPs of 5 mg kg^−1^ into ICR mice via the tail vein to further assess the biocompatibility of the SAMPs *in vivo*. Analysis of blood parameters, as shown in Fig. 9E through L, revealed no significant differences in RBC, WBC, PLT, HCT, HGB, MCV, MCH, and MCHC between the PBS and SAMPs groups, and the blood routine indices remained within normal physiological ranges, indicating the absence of acute and chronic hematologic toxicity induced by SAMPs. These results demonstrated that the potential side effects of SAMPs are minimal, highlighting their excellent biocompatibility *in vivo*.

### *In vivo* sepsis efficacy evaluation of SAMPs

**Fig 10:**
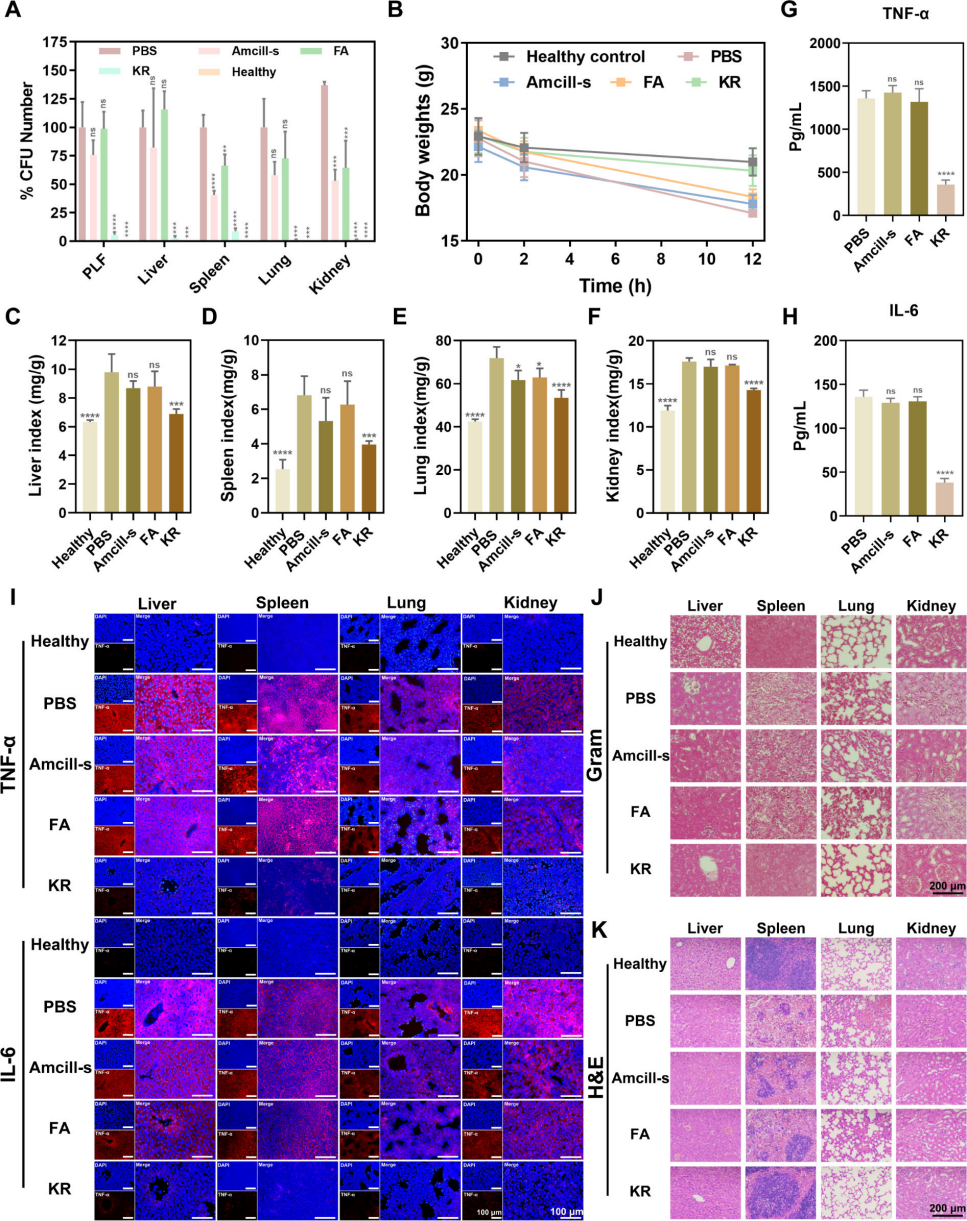
*In vivo* sepsis efficacy evaluation of SAMPs. (**A**) Bacterial quantification in the MDR-*E. coli*-infected liver, spleen, lung, kidney, and PLF after treatment with PBS, FA, Amcill-s, or KR (5 mg kg^-1^). (**B**) Body weight changes of mice during treatment. Relative organ index (organ mass/weight of mice before execution) of (**C**) liver, (**D**) spleen, (**E**) lung, and (**F**) kidney after treatment. The (**G**) TNF-α and (**H**) IL-6 levels in mouse serum after treatment. Data are presented as mean ± SD (*n* = 6); *P < 0.05, **P < 0.01, ***P < 0.001, ****P < 0.005. (**I**) Immunofluorescence (TNF-α and IL-6) images of liver, spleen, lung, and kidney after treatment, Scale bar: 100 μm. (**J**) Gram-stained images and (**K**) H&E-stained images of liver, spleen, lung, and kidney after treatment, Scale bar: 200 μm.

Sepsis is a systemic inflammatory response induced by bacterial infection with a high mortality rate (8). To assess the efficacy of SAMPs in combating sepsis, particularly in response to MDR-*E. coli* infection, a sepsis model was established. After post-infection of 2 h, the safe dose of FA and KR (5 mg kg^−1^) was administered intraperitoneally to mice, with PBS serving as a positive control and Amcill-s (5 mg kg^−1^) as an antibiotic control. After 12 h of infection, mice were euthanized, and the number of bacterial colonies present in the organs was quantified. As shown in Fig. 10A, the bacterial burden in the liver, spleen, lung, kidney tissues, and peritoneal lavage fluids (PLF) was significantly decreased after treatment with KR compared to other treatments, which indicates that the potent antimicrobial ability and stability of KR were maintained at the level *in vivo*. Simultaneously, we monitored the weight changes in mice pre- and post-treatment and found that mice treated with KR exhibited stable weight comparable to that of healthy mice (Fig. 10B), with slight weight loss attributable to fasting. However, mice in other infected groups experienced greater weight loss, indicating the safety and efficacy of KR treatment. By means of further weighing the organs of mice and calculating the relative organ index, Fig. 10C through F revealed varying degrees of swelling in the liver, spleen, lung, and kidney due to bacterial infection (evidenced by increased mass). But the KR group returned to normal size with a significant decrease in the organ relative index, approaching that of healthy mice. Notably, significantly lower levels of pro-inflammatory factors, such as tumor necrosis factor-α (TNF-α) and interleukin (IL)-6, were observed in the serum (Fig. 10G and H) and organs (Fig. 10I) of mice treated with KR compared to those treated with PBS, FA, and Amcill-s. These pro-inflammatory factors are known to be involved in the inflammatory response during acute sepsis, indicating that SAMPs may possess immunomodulatory properties to assist in antimicrobial activity. Additionally, the histopathological assessments were conducted to evaluate the impact of bacterial burden on tissue changes. Gram-stained images of liver, spleen, lung, and kidney showed a reduction in MDR-*E. coli* staining following treatment with KR (Fig. 10J), which was consistent with the above colony quantification results. Histological hematoxylin−eosin (H&E) images in Fig. 10K demonstrated broad pathological alterations in the tissues of infected mice, such as hepatocyte damage, red pulp atrophy, inflammatory cell infiltration, and abnormal glomerulus structure. In contrast, the KR group exhibited substantial restoration of tissue injuries, similar to that of the healthy group. Taken together, these findings underscore the potential of the KR in mitigating sepsis and bacterial infections by reducing the bacterial burden, regulating inflammatory factor disorders, and attenuating tissue injury.

### Conclusion

In summary, this study reports the design of a series of antibacterial and antibiofilm SAMPs by rational database-filtering technology, modifying structure–function relationships. The potential applications of these SAMPs *in vivo* and *in vitro*, as well as their stability, biosafety, and antibacterial mechanisms of these SAMPs, were thoroughly investigated. These SAMPs display varying levels of inhibitory and bactericidal activity against Gram-negative and Gram-positive bacteria and their biofilms while maintaining reliable biosafety and stability both *in vivo* and *in vitro*, without inducing drug resistance. In the treatment of sepsis caused by acute bacterial infection of MDR-*E. coli*, the use of SAMPs relieved the organ bacterial burden and inflammatory factor levels of infected mice. Furthermore, the multimodal antimicrobial mechanisms of SAMPs were found to encompass membrane depolarization and increased permeability, loss of cellular contents, induction of an apoptotic-like death pathway accompanied by ROS production, and interference with ribosome biogenesis, energy production, and other normal physiological processes, ultimately resulting in bacterial cell death. Collectively, this study provided a reference approach for peptide engineering to facilitate the development of SAMPs against Gram-negative bacteria in clinical settings.

## MATERIALS AND METHODS

### Materials

10× PBS solution was procured from BioSharp (China); yeast extract and tryptone was obtained from Thermo Fisher (USA); Agar powder, sodium dodecyl sulfate (SDS), ethylenediaminetetraacetic acid disodium salt (EDTA-2Na), Triton X-100, ampicillin sodium, kanamycin sulfate, crystal violet, PI solution, 10× DNA loading buffer, 2-(4-Amidinophenyl)-6-indolecarbamidine dihydrochloride (DAPI), calcein-AM/PI Dead/Live Cell Dual Staining Kit and Annexin V-FITC Apoptosis Detection Kit were obtained from Solarbio (China); 3,3'′-dipropylthiadicarbocyanine Iodide (DiSC_3_-5) was purchased from AAT BioQuest (USA); HEPES buffer was purchased from Yuanye (China); methanol, 25% aqueous glutaraldehyde solution, absolute ethanol, sodium chloride (NaCl), and potassium chloride (KCl) were obtained from Sinopharm Chemical Reagent Co., Ltd. (China); TFE was obtained from Sigma Aldrich (USA); 2′,7′-dichlorodihydrofluorescein diacetate (DCFH-DA) and dimethyl sulfoxide (DMSO) were obtained from J&K Chemical Technology (China); Micro-MDA Assay Reagent Kit was purchased from KeyGEN Biotech (China); Cell Counting Kit-8 (CCK8) was purchased from GlpBio (USA); Bacterial Genomic DNA Extraction Kit was purchased from TIANGEN (China); Bacteria RNA Extraction Kit, HiScript III RT SuperMix for qPCR(+gDNA wiper) and Taq Pro Universal SYBR qPCR Master Mix were obtained from Vazyme (China); 2 kb DNA ladder (100-2000 bp) was obtained from Baiaolaibo (China); Mouse TNF-α ELISA kit and Mouse IL-6 ELISA kit were obtained from Shanghai Enzyme-linked Biotechnology Co., Ltd. (China); Anti-IL-6 antibody (ab233706) and goat Anti-rabbit IgG H&L (ab150078, Alexa Fluor 555) were obtained from Abcam (UK); Anti-TNF-α (D2D4) XP rabbit monoclonal antibody was obtained from Cell Signaling Technology (USA); All the mice were purchased from Jinan Pengyue Experimental Animal Breeding Co., Ltd. (China) and kept in a specific pathogen-free environment.

### Peptide synthesis

The seven SAMPs listed in Table 1 were synthesized by the standard solid-phase FMOC method at GL Biochem (Shanghai, China) Ltd., with amidation of the C-terminus. 5-Carboxyl TMR fluorescein-modified KR (TMR-KR) was synthesized by Bioengineering Co., Ltd. (Shanghai, China). All SAMPs were purified after synthesis using high-performance liquid chromatography with a purity greater than 95%, and the molecular weights were identified using mass spectrometry (MS). All SAMP samples were stored at -20°C.

### Structural parameters prediction of the SAMPs

The three-dimensional structure modeling of the SAMPs was predicted online utilizing the I-TASSER (http://zhanglab.ccmb.med.umich.edu/I-TASSER/). The secondary structure projection was performed using the protein secondary structure prediction website (https://www.novopro.cn/tools/secondary-structure-prediction.html). The helical wheel projection of the SAMPs was calculated using the online program NetWheels Peptides Helical Wheel and the Net projection maker (http://lbqp.unb.br/NetWheels/). The molecular weights of all the SAMPs were calculated online with the ExPASy Proteomics Server (https://web.expasy.org/peptide_mass/). The net charge and hydrophobic content of SAMPs were calculated using the APD (https://aps.unmc.edu/prediction).

### CD spectroscopy detection of the SAMPs

The SAMPs were dissolved to 150 μM in PBS (10 mM, pH 7.4, 1 mL), SDS (30 mM, 1 mL), and TFE (50%, vol vol^−1^, 1 mL) solution to mimic the normal physiological, hydrophobic, and microbial membrane environments, respectively. CD spectra of SAMPs were recorded using a spectrometer (Jasco J-1500, Tokyo, Japan) in a quartz cuvette with a 1.0 mm path length, scanning from 195 to 250 nm at a speed of 10 nm min^−1^. Scanning was repeated thrice, and peak plots were generated using GraphPad Prism 7.

### Bacterial cultivation

Two MDR bacteria, MDR-*E. coli* (LZ-7, drug resistance: compound sulfamethoxazole, ampicillin, gentamicin, ciprofloxacin, levofloxacin) and MDR-*A. baumannii* (AB-29, drug resistance: 11 kinds of antibiotics including cephalosporins), along with three pathogenic bacteria, *E. coli* (ATCC 25922), *S. aureus* (ATCC 6538), and *A. baumannii* (309-14) were included in the study. A single colony of each bacterium was cultured in Luria-Bertani (LB) medium at 37°C and 180 rpm for 12–16 h until the logarithmic growth phase for use.

### Antimicrobial activity assay

The MIC of the peptides against a range of bacteria was determined using the microbroth dilution method with slight modifications (62). In brief, the bacteria mentioned above were dispersed in the LB (10^5^ CFU mL^−1^) and were mixed with gradient concentrations (0.25 μM–16 μM) of SAMPs in the 96-well plate (*n* = 3). After incubation at 37°C for 16–18 h, the absorbance at 600 nm was measured using a microplate reader (Thermo Fisher Scientific, Multiskan MK3, China). The lowest peptide concentration with no absorbance increase was defined as the MICs. The bacterial solution in the 96-well plate was then transferred to solid LB medium to culture single colonies (*n* = 3), and the lowest peptide concentration at which almost no colonies survive (less than 0.01%) was defined as the MBCs.

### Kill-time kinetics assay

The antimicrobial activity kinetics was assessed using the plate colony counting method. (63) In brief, bacterial suspensions of *S. aureus* and *E. coli* (10^5^ CFU mL^−1^) were incubated with SAMPs (1× MBCs) at 37°C for varying time intervals (1 min, 5 min, 10 min, 15 min, 20 min, 25 min, and 30 min). Untreated bacterial samples served as the control. Subsequently, the bacterial samples were plated on LB agar (*n* = 3), and colonies were counted after overnight incubation at 37°C.

### Salt and serum sensitivity assay

Based on previous methods, stability was analyzed by monitoring the changes in the antimicrobial efficacy of SAMPs in the presence of salts and serum (37, 38). The SAMPs underwent incubation with physiological salts (150 mM NaCl, 4.5 mM KCl) or serum at 37°C for 12 h, followed by measurement of their MICs using the previously outlined method (*n* = 3).

### Thermal and acid-base stability assay

This experimental procedure was conducted in accordance with a modified version of a previously established method (64). Simply put, the SAMPs were subjected to pre-treatment at elevated temperatures (60°C, 80°C) for 0.5 h, or pre-treated with PBS (pH 5.5, pH 8.5) for 2 h at 37°C, respectively, after which the MICs of the treated SAMPs were determined using the same methodology as described above (*n* = 3).

### Inhibitory of bacterial biofilm formation assay

The inhibitory effect of AMPs on bacterial biofilm growth was assessed by previous crystal violet staining methods (65). Bacterial cultures of *S. aureus* and *E. coli* (10^8^ CFU mL^−1^) in LB were inoculated into 24-well plates (1 mL per well, *n* = 3) and incubated at 37°C for 24 h. After being washed with sterile PBS, the preformed biofilms of *S. aureus* and *E. coli* were exposed to SAMPs (8 μM) in LB medium or blank LB medium at 37°C for 48 h. The LB medium was replaced every 12 h. Then, the medium was removed and the plates were washed with sterile PBS. The biofilms were fixed with methanol for 15 min at 4°C. Afterward, the biofilms were fully washed and stained with 0.1% crystal violet dye solution for 30 min. After being fully washed twice with PBS and dried at 37°C, the biofilms were photographed and recorded. Then, anhydrous ethanol was added to each well and incubated for 30 min at room temperature with shaking. The supernatant was added to 96-well plates, and the OD_550_ nm values were read using a microplate reader (Thermo Fisher Scientific, Multiskan MK3, China).

### Destruction of bacterial biofilm assay

The culture of bacterial biofilms is the same as above, and the destructive effect of SAMPs on bacterial biofilms was observed by using Dead/Live staining according to previous literature (66). To put it simply, bacterial suspensions of *S. aureus* and *E. coli* (10^8^ CFU mL^−1^) in LB were added into confocal dishes (1 mL per well, *n* = 3) and incubated at 37°C for 5 days to form complete biofilms. The medium was changed every 12 h, and after being washed with sterile PBS, the biofilms were exposed to seven SAMPs (8 μM) or blank LB medium at 37°C for 4 h. Then, the bacterial biofilms were subjected to staining with the Calcein-AM/PI Dead/Live double staining kit (Solarbio, CA1630) in the dark. After 20 min, the stained bacterial biofilms were thoroughly rinsed with sterile PBS and visualized using a 3D CLSM (Nikon, ECLIPSE Ti2-E, Japan).

### Intracellular localization of SAMPs

Bacterial suspensions of *S. aureus*, *E. coli*, *A. baumannii*, MDR-*E. coli,* and MDR-*A. baumannii* at a concentration of 10^5^ CFU mL^-1^ were prepared and then incubated with KR modified by fluorophore TMR (0.2 μM) at 37°C for 1, 10, and 30 min in dark (*n* = 3). Subsequently, bacterial suspensions were concentrated to 10^8^ CFU mL^-1^ and stained with DAPI dye (500 μL, 10 μg mL^-1^) at room temperature for 5 min, followed by washing through centrifugation and resuspension in PBS for immediate observation under a CLSM (Nikon, ECLIPSE Ti2-E, Japan). In addition, the intracellular fluorescence intensity of the bacterial samples was quantified by a microplate reader (Thermo Fisher Scientific, Multiskan MK3, China).

### SEM and TEM characterization of bacterial morphology

Bacterial suspensions of *S. aureus* and *E. coli* (10^8^ CFU mL^−1^) were treated with seven SAMPs (2 μM) or PBS at 37°C for 30 min (*n* = 3). Centrifugation at 3,000 rpm for 5 min was used to collect bacterial cells. Then the bacterial cells were fixed with glutaraldehyde (2.5% wt vol^−1^) at 4°C for a period of 12 h. According to established protocols, the fixed bacterial cells were prepared into SEM (67) and TEM (3) samples, respectively, and then observed with SEM (FEI, Nova nanoSEM 450) and TEM (JEOL, JSM-840).

### CM depolarization assay

The depolarization activity of the SAMPs on the CM was evaluated by utilizing the membrane-potential-sensitive fluorescent probe DiSC_3_-5. Bacterial suspensions of *S. aureus*, *E. coli*, *A. baumannii*, MDR-*E. coli,* and MDR-*A. baumannii* were diluted to 10^6^ CFU mL^−1^ in a 5 mM HEPES buffer (pH 7.4, containing 100 mM KCl) and incubated with an equal volume of DiSC_3_-5 (0.5 μM) for 30 min in the dark (*n* = 3). Then, the bacterial samples were mixed with SAMPs (2 μM), with the bacterial samples lacking SAMPs serving as the negative control. Fluorescence measurements were taken at excitation and emission wavelengths of 620 nm and 670 nm, respectively, and recorded for 30 min using a microplate reader (PerkinElmer, Enspire2300, USA) immediately.

### Membrane permeability assay

The alteration in cell membrane permeability was assessed via flow cytometry. Briefly, the bacterial suspensions of *S. aureus*, *E. coli*, *A. baumannii*, MDR-*E. coli,* and MDR-*A. baumannii* (10^5^ CFU mL^-1^) were treated with seven SAMPs (0.2 μM) or PBS at 37°C for 30 min (*n* = 3). Subsequently, the bacterial samples underwent a washing step with PBS and were then stained with a PI solution (10 μL mL^-1^, 1 mL) for 30 min in the dark. Following a subsequent washing step with PBS, the intracellular fluorescence was measured using a FACS flow cytometer (Beckman Coulter, FC500 MPL, USA).

### DNA-binding affinity assay

The DNA gel retardation method previously described was employed for this experiment (64). Briefly, the genomic DNA from *S. aureus*, *E. coli*, *A. baumannii*, MDR-*E. coli,* and MDR-*A. baumannii* (10^8^ CFU mL^−1^) was extracted using a bacterial genomic DNA extraction kit (TIANGEN, China). Then, DNA was incubated with SAMPs (2 μM) at 37°C, with DNA samples lacking SAMPs serving as the control. Following a 30-min treatment period, the genomic DNA bands were identified by agarose gel electrophoresis using Gel Red staining.

### Cell cycle assay

Bacterial suspensions were prepared using the same method as 4.15 for cell cycle analysis (68). Briefly, the bacterial suspensions of *S. aureus* and *E. coli* (10^5^ CFU mL^−1^) were treated with seven SAMPs (0.2 μM) or PBS at 37°C for 30 min (*n* = 3). After being washed with PBS, the bacterial cells were fixed with 70% ethanol at 4°C overnight. Subsequently, the bacterial samples were washed and resuspended in a dye solution containing PI (20 μg mL^−1^) and ribonuclease (RNase, 200 μg mL^−1^) and incubated in the dark for 20 min. After being washed with PBS, the cell cycle was analyzed using a FACS flow cytometer (Beckman Coulter, FC500 MPL, USA).

### Cell apoptosis assay

Apoptosis was assessed using a modified method as previously described (49). The bacterial suspensions of *E. coli* (10^5^ CFU mL^−1^) were treated with seven SAMPs (0.2 μM) or PBS at 37°C for 30 min (*n* = 3). After being washed with PBS, the bacterial samples were stained with an Annexin V-FITC Apoptosis Detection Kit (Solarbio, CA1020, China) and analyzed using a FACS flow cytometer (Beckman Coulter, FC500 MPL, USA).

### PCD pathways assay

The relative mRNA expression levels of RecA and LexA were quantified using quantitative real-time PCR (qRT-PCR) to investigate PCD pathways (47). Bacterial suspensions of *E. coli* (10^5^ CFU mL^−1^) were incubated with FA, KR (0.2 μM), or PBS at 37°C for 4 h and then were adjusted to 10^8^ CFU mL^−1^ (*n* = 3). Following pre-treatment with a Bacteria RNA Extraction Kit (Vazyme, China), total RNA was extracted from *E. coli* using the TRIzol method (69). Then the total RNA was reverse-transcribed to complementary DNA using a HiScript III RT SuperMix for qPCR (+gDNA wiper) Kit. RT-PCR was conducted using a real-time PCR system (Eppendorf, Germany) and Taq Pro Universal SYBR qPCR Master Mix (Vazyme, China). The gene expression was normalized to the corresponding 16S rRNA level, and the following primers were used: recA (For) AGATCCTCTACGGCGAAGGT, (rev) CCTGCTTTCTCGATCAGCTT; lexA (For) GACTTGCTGGCAGTGCATAA, (rev) TCAGGCGCTTAACGGTAACT; 16SrRNA (For) TGTAGCGGTGAAATGCGTAGA, and (rev) CACCTGAGCGTCAGTCTTCGT (70). All the primers were synthesized by Sangon Biotech Co., Ltd. (Shanghai). The relative fold change of mRNA expression was calculated according to the 2^−ΔΔCt^ method (71).

### Determination of intracellular ROS

ROS levels in bacteria were detected using 2′,7′-dichlorofluorescein diacetate (DCFH-DA) (72). Briefly, the bacterial suspensions of *S. aureus*, *E. coli*, *A. baumannii*, MDR-*E. coli,* and MDR-*A. baumannii* (10^5^ CFU mL^−1^) were treated with seven SAMPs (0.2 μM) or PBS at 37°C for 30 min after being stained with DCFH-DA (10 mM) at 37°C for 30 min (*n* = 3). Following treatment, the bacterial samples were thoroughly washed with PBS, and the fluorescence intensity at an excitation wavelength of 488 nm and an emission wavelength of 525 nm was determined immediately using a microplate reader (Thermo Fisher Scientific, Multiskan MK3, China).

### Determination of intracellular MDA

The level of intracellular lipid oxidation was assessed using MDA as a marker (73). The bacterial suspensions of *E. coli* (10^5^ CFU mL^−1^) were treated with seven SAMPs (0.2 μM) or PBS at 37°C for 4 h (*n* = 3). Following ultrasonic disruption, the bacterial samples were suspended in PBS for the assessment of lipid peroxidation levels using a Micro-MDA Assay Reagent Kit (KeyGEN Biotech, China).

### RNA isolation and transcriptome analysis

The bacterial suspensions of *E. coli* (10^5^ CFU mL^−1^) were incubated with KR (0.2 μM) or PBS at 37°C for 4 h (*n* = 3). Subsequently, the bacterial samples were washed with PBS and adjusted to 10^8^ CFU mL^−1^, and then stored at −80°C. The RNA extraction, library construction, and sequencing analysis were entrusted to Majorbio Bio-Pharm Technology Co., Ltd. (Shanghai). Four independent samples were contained in each group. The DESeq2 R software package was used for statistical analysis, and a negative binomial distribution corrected *P* value < 0.05 and |log_2_(fold change)| > 1 were regarded as the thresholds for significant differential expression. KEGG enrichment analysis was carried out using the hypergeometric distribution and false-discovery rate correction.

### Drug resistance assay

The evaluation of drug resistance in SAMPs was performed through MIC value detection (74). Resistance was defined as a greater than fourfold increase in the MIC from its initial value. *S. aureus* and *E. coli* grown in LB medium supplemented with 0.5 × MIC of each SAMP were repeated for 30 days, followed by determination of MIC values using the same methodology outlined above (*n* = 3). Meanwhile, Colistin E (4 μg mL^-1^) was used as the control group to induce bacterial resistance in the same way.

### Cytotoxicity assay

*In vitro* cytotoxicity against RAW 264.7 cells was investigated by using an established Cell Counting Kit 8 (CCK-8) assay (75). Specifically, RAW 264.7 cells were cultured in high-glucose Dulbecco’s modified Eagle’s medium (DMEM) supplemented with 10% fetal bovine serum and 1% antibiotics (37°C, 5% CO_2_). After reaching the logarithmic phase, RAW264.7 cells were inoculated into 96-well plates at a density of 10^4^ cells per well (*n* = 3). After incubation overnight, DMEM medium containing different concentrations of SAMPs (0.25–2 μM, 100 μL) was added to wells and incubated at 37°C for 4 h. The fresh DMEM medium without SAMPs was added as a control. At predetermined exposure times, each well was washed and supplemented with 100 μL fresh DMEM medium containing 10 μL of CCK-8 reagent (Glpbio, USA), and the cells were further incubated for 4 h at 37°C. The absorbance (A) at 450 nm was measured using a microplate reader (Thermo Fisher Scientific, Multiskan MK3, China). Cell viability was calculated using the formula: Cell viability (%) = [(*A* − *A*_0_)/(*A*_c_− *A*_0_)] × 100 (%).

### Hemolysis assay

The hemolytic activity of the SAMPs was assessed following established protocols (76). Fresh mouse red blood cells (hRBCs) were washed thrice and diluted in PBS (pH 7.4) to acquire a suspension (2%, vol vol^−1^). Then different concentrations of SAMPs (0.25–2 μM), Triton X-100 (2%, vol vol^−1^), and PBS were individually combined with an equal volume (350 μL) of hRBCs solution and incubated for 1 h at 37°C (*n* = 3). After incubation, the supernatant was isolated, photographed, and transferred to a 96-well plate. The absorbance (A) at 570 nm was measured using a microplate reader (Thermo Fisher Scientific, Multiskan MK3, China). Hemolysis was calculated using the formula: Hemolysis (%) = [(*A* − *A*_0_)/(*A*_c_− *A*_0_)] × 100 (%).

### *In vivo* toxicity assay

All animal experiments were conducted in accordance with the guidelines set forth by the National Research Council Guide (1996). Healthy female ICR mice (20 ± 2 g) were randomly divided into eight groups with three replicates per group. The mice were administered daily intravenous injections of 100 μL of seven SAMPs (5 mg kg^-1^) or PBS for two consecutive days via the tail vein. Blood samples were collected from the orbital vein on the first and seventh days after injection and mixed with an anticoagulant. An automated hematology analyzer (MINDRAY, BC-2800Vet, China) was used for hematological index detection.

### *In vivo* therapeutic effect evaluation

All animal experiments were conducted in accordance with the guidelines set forth by the National Research Council Guide (1996). Healthy female ICR mice (20 ± 2 g) were randomly divided into five groups with six replicates per group. The four group mice were infected by injecting MDR-*E. coli* (OD_600_ = 0.3, 100 μL) into the peritoneal cavity to induce sepsis, while the mice in the healthy control group were injected with PBS. Two hours post-infection, the infected mice in all four groups were administered intraperitoneal injection of 100 μL of PBS, Amcill-s (5 mg kg^−1^), FA (5 mg kg^−1^), and KR (5 mg kg^−1^). After 12 h, all mice were euthanized, and their weight and survival rates were documented. Blood was drawn from the orbital vein of the mice for the assessment of inflammatory factors in the serum, such as TNF-α and IL-6, using commercially available diagnostic kits (Shanghai Enzyme-linked, China). Additionally, the liver, kidney, spleen, and lung of the mice were isolated and weighed. Two mice from each group were allocated for H&E and Gram staining, two for immunofluorescence staining (IL-6 and TNF-α), and the remaining mice were used for a quantitative bacterial analysis.

### Statistical analysis

Quantitative data were presented as the mean ± standard deviation (SD) using GraphPad Prism 7 software. Statistical significance was assessed through an unpaired t-test using the same software, with significance levels denoted as *P < 0.05, **P < 0.01, ***P < 0.001, and ****P < 0.005 considered statistically significant.

## SUPPLEMENTAL MATERIAL

Supplemental material aac.01343-25-s0001.docx Fig. S1 to S29; Table S1.

ASM does not own the copyrights to Supplemental Material that may be linked to, or accessed through, an article. The authors have granted ASM a non-exclusive, world-wide license to publish the Supplemental Material files. Please contact the corresponding author directly for reuse.
