# Supplementary material for: A short antimicrobial peptides family demonstrates efficacy to infection via a multimodal mechanism of action
Source: Antimicrob Agents Chemother. 2025 Dec 23;70(2):e01343-25. doi: 10.1128/aac.01343-25 (PMC12888869; doi:10.1128/aac.01343-25)
Supplement: Supplemental material — Fig. S1 to S29; Table S1. [file aac.01343-25-s0001.docx]

**Supporting Information**

**A Short Antimicrobial Peptides Family Demonstrates Efficacy to Infection via a Multimodal Mechanism of Action**

*Yifan Liu^1，2^, Pengfei Cui^1^*, Jingyi Sun^3^, and Shaoguo Ru^1^**

^1^ Lab of Environmental Health and ecological engineering, College of Marine Life Science, Ocean University of China, Qingdao 266003, China

^2^ Jiangsu Medical College, Yancheng, 224005, China

^3^ North China Sea Marine Forecasting Center of State Oceanic Administration, Qingdao 266003, China

E-mail: [rusg@ouc.edu.cn](mailto:rusg@ouc.edu.cn); [cuipengfei@ouc.edu.cn](mailto:cuipengfei@ouc.edu.cn)


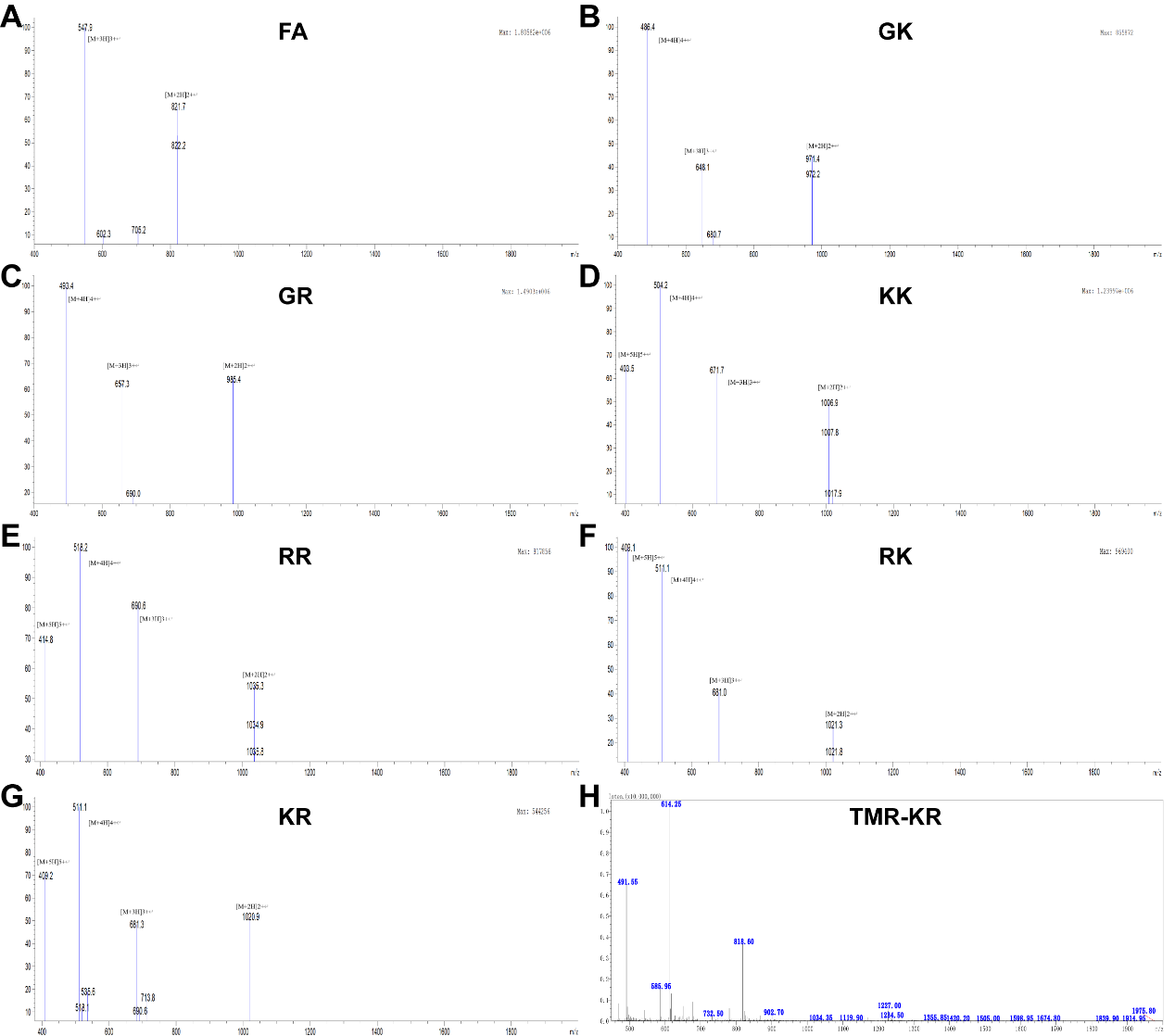


**Figure S1**. MS results of (A) FA, (B) GK, (C)GR, (D) KK, (E)RR, (F)RK, (G)KR and (H) TMR-KR.


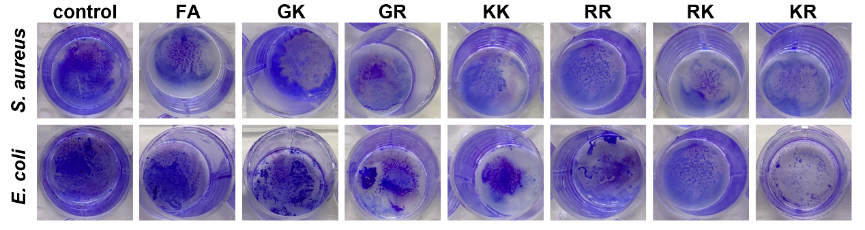


**Figure S2**. Photographs of crystal violet stained *S. aureus* and *E. coli* biofilms after treatment with SAMPs (8 μM).


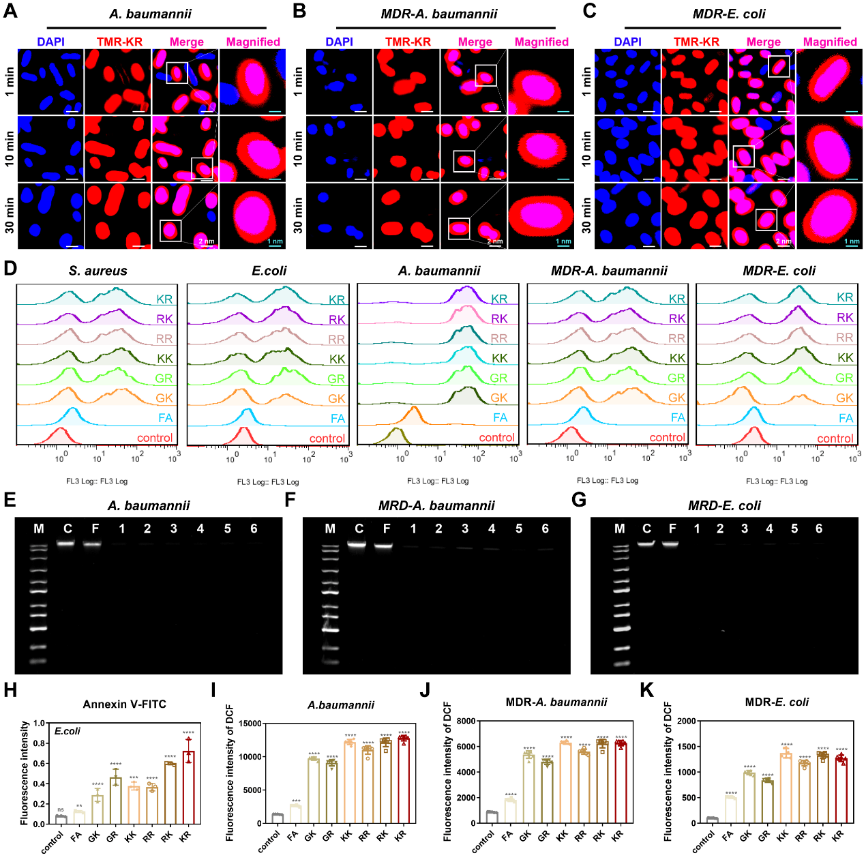


**Figure S3**. Confocal laser microscope photos of DAPI-stained (A) *A. baumannii*, (B) MDR-*A. baumannii* and (C) MDR-*E. coli* treated with TMR-KR (0.2 μM) for 1, 10, and 30 min. (Red: TMR-KR, excitation at 558 nm, emission at 586 nm; Blue: DAPI, excitation at 340 nm and emission at 488 nm; Pink: Merge, the overlap of red fluorescence and blue fluorescence). White scale bar: 2 µm, green scale bar: 1 µm. (D) Cytoplasmic membrane permeability of *S. aureus*, *E. coli*, *A. baumannii*, MDR-*E. coli* and MDR-*A. baumannii* induced by SAMPs (0.2 μM); excitation wavelength 535 nm, emission wavelength 615 nm. Genomic DNA degradation detection of (E) *A. baumannii*, (F) MDR-*A. baumannii* and (G) MDR-*E. coli* treated with SAMPs (2 μM). M, DNA Maker; C, blank control; F, FA;1, GK; 2, GR; 3, KK; 4, RR; 5, RK; 6, KR. (H) Cell apoptosis detection of *E. coli* treated with SAMPs (0.2 μM). Fluorescence intensity quantification of (I) *A. baumannii*, (J) MDR-*A. baumannii* and (K) MDR-*E. coli* staining by DCFH-DA after treatment with SAMPs (0.2 μM). Data are presented as mean ± SD (n = 3); ** means p < 0.01, *** means p < 0.001, **** means p < 0.005, and ns means no significant difference.


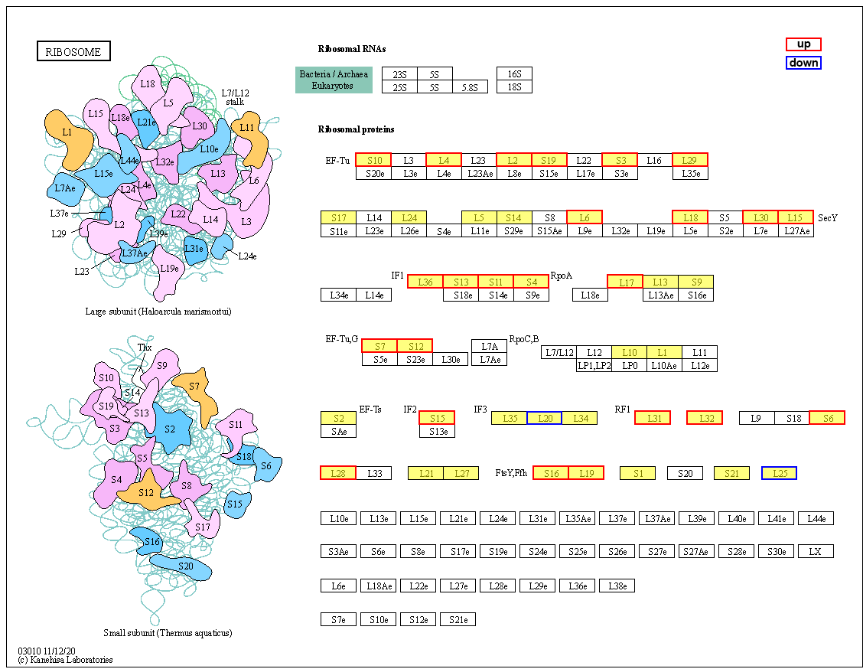


**Figure S4**. KEGG analysis of ribosome in *E. coli* treated with KR. Red boxes indicate significantly upregulated genes, and blue boxes indicate significantly downregulated genes.


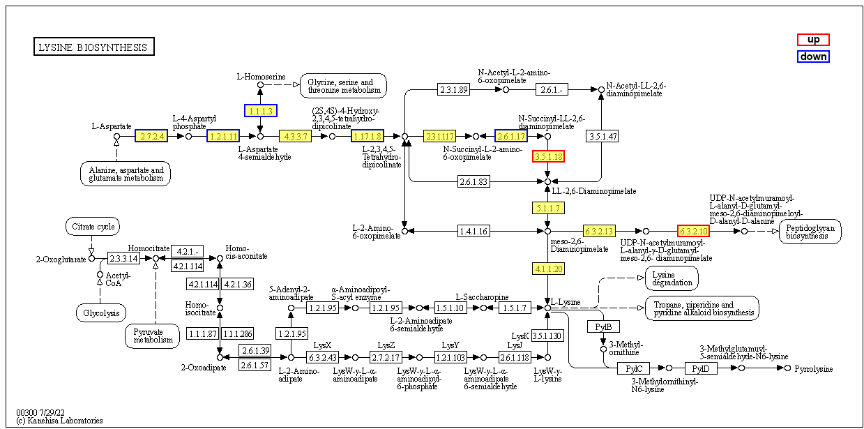


**Figure S5**. KEGG analysis of lysine biosynthesis in *E. coli* treated with KR. Red boxes indicate significantly upregulated genes, and blue boxes indicate significantly downregulated genes.


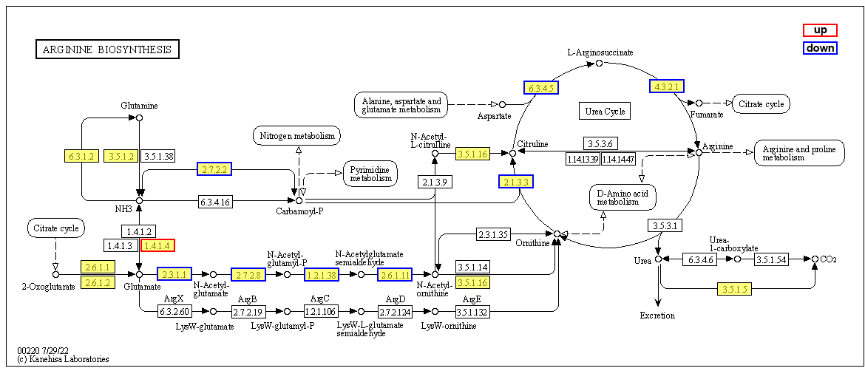


**Figure S6**. KEGG analysis of arginine biosynthesis in *E. coli* treated with KR. Red boxes indicate significantly upregulated genes, and blue boxes indicate significantly downregulated genes.


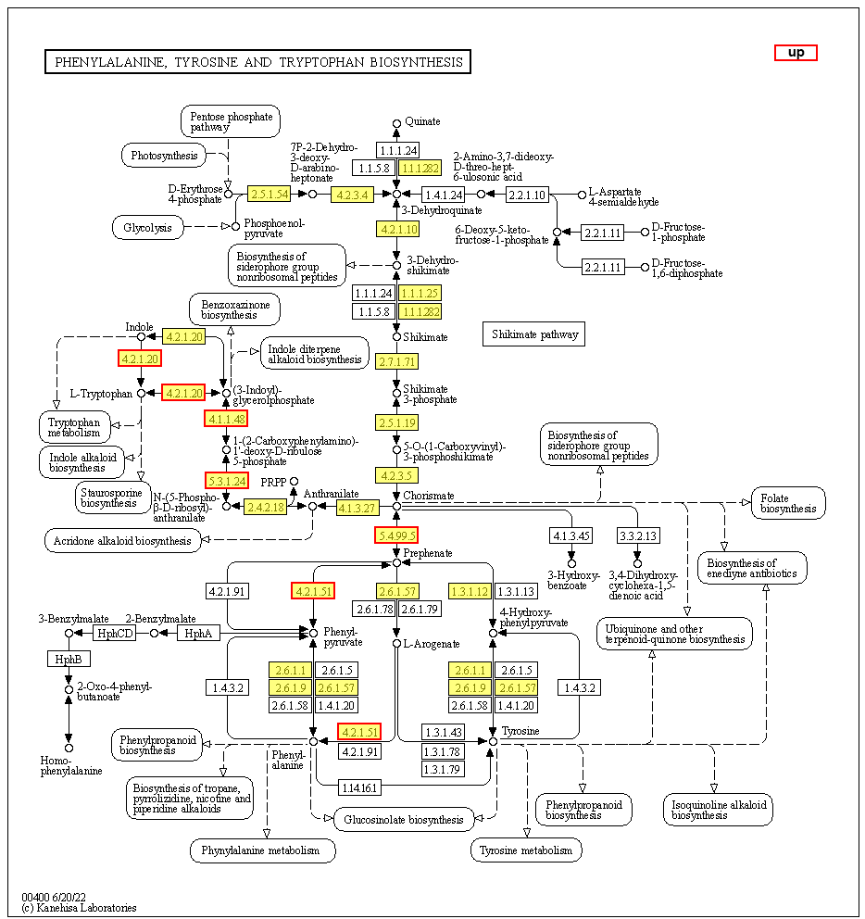


**Figure S7**. KEGG analysis of phenylalanine, tyrosine and tryptophan biosynthesis in *E. coli* treated with KR. Red boxes indicate significantly upregulated genes, and blue boxes indicate significantly downregulated genes.


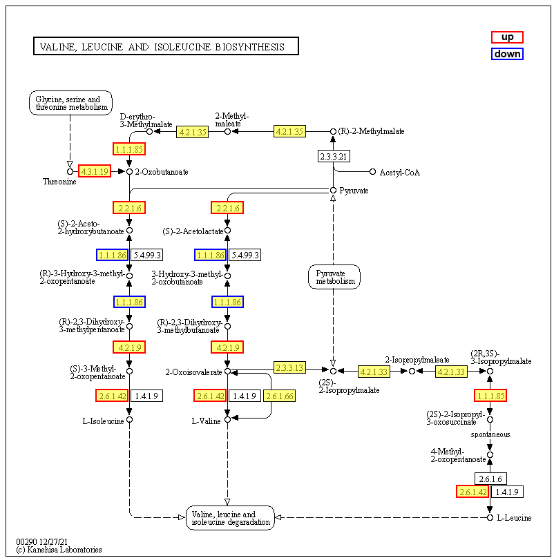


**Figure S8**. KEGG analysis of valine, leucine and isoleucine biosynthesis in *E. coli* treated with KR. Red boxes indicate significantly upregulated genes, and blue boxes indicate significantly downregulated genes.


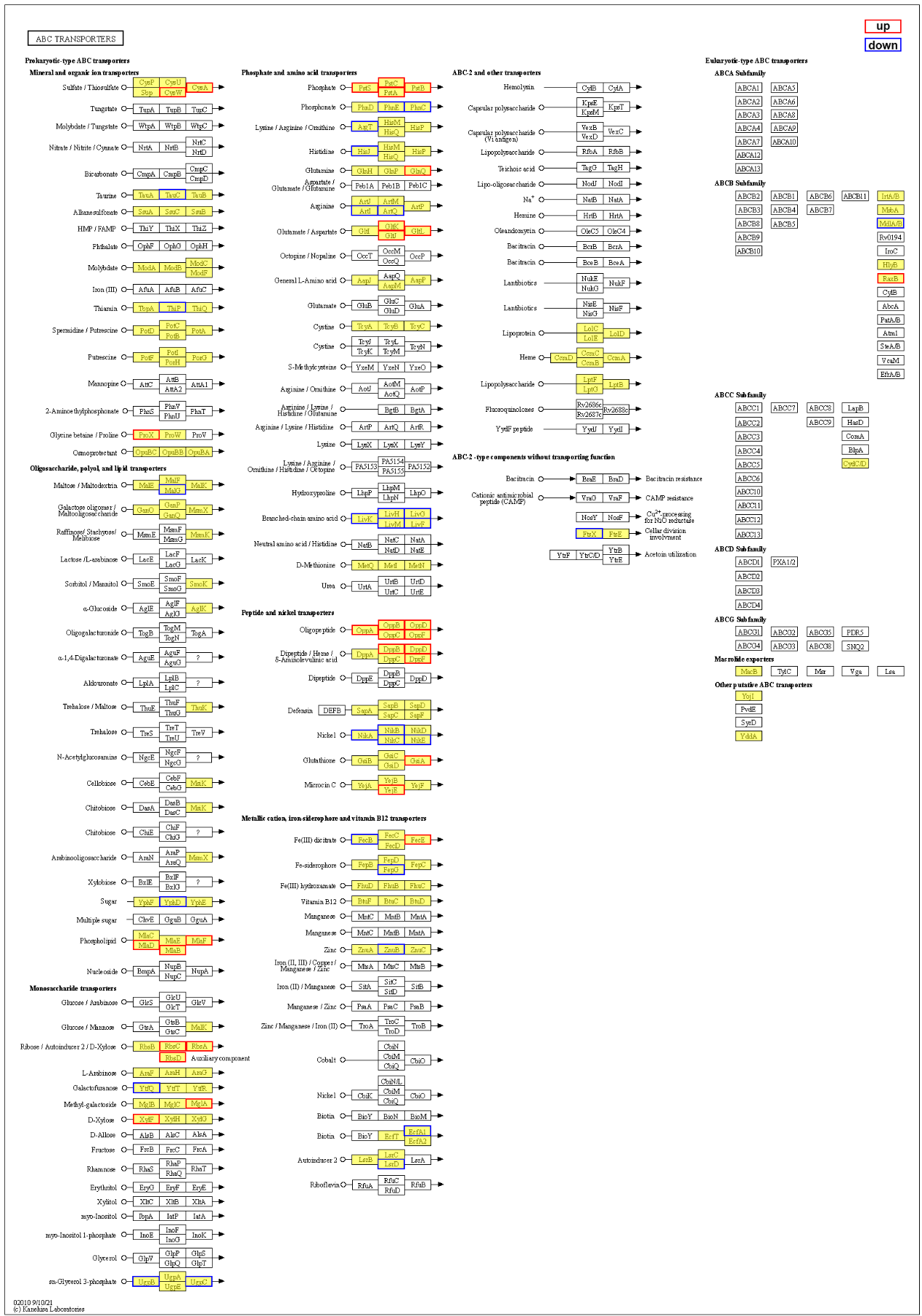


**Figure S9**. KEGG analysis of ABC transporter in *E. coli* treated with KR. Red boxes indicate significantly upregulated genes, and blue boxes indicate significantly downregulated genes.


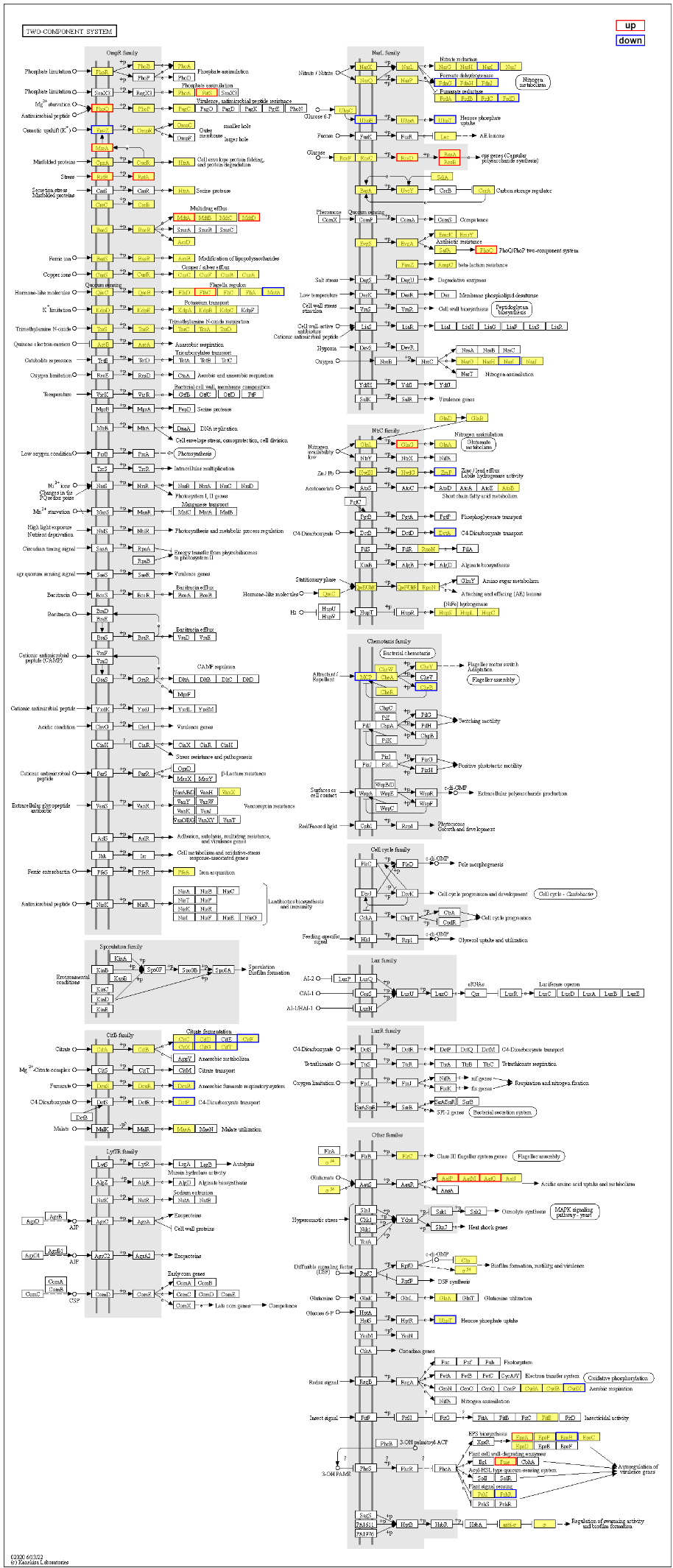


**Figure S10**. KEGG analysis of two-component system in *E. coli* treated with KR. Red boxes indicate significantly upregulated genes, and blue boxes indicate significantly downregulated genes.


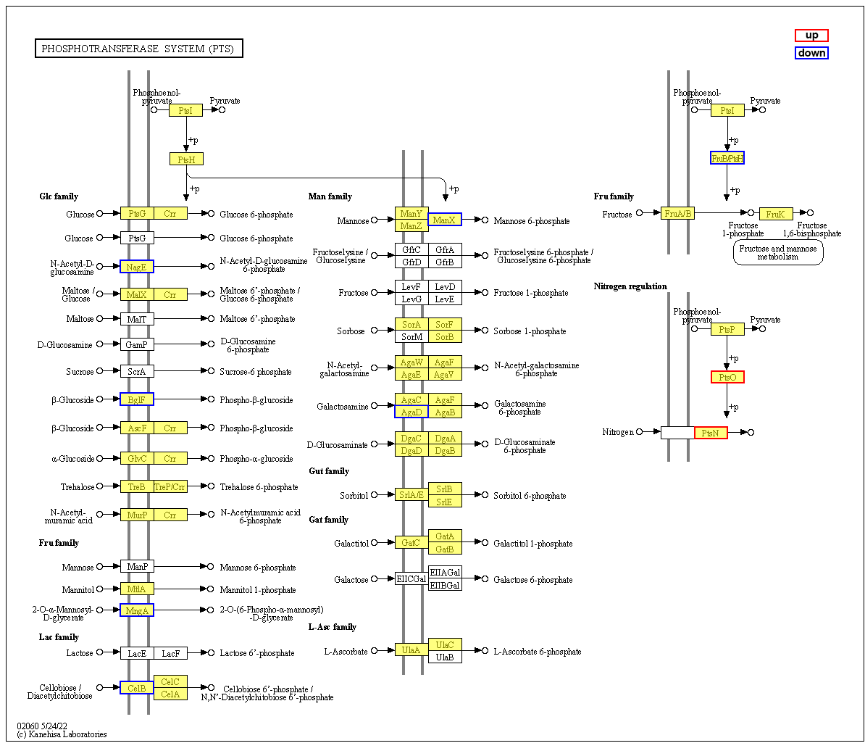


**Figure S11**. KEGG analysis of phosphotransferase system (PTS) in *E. coli* treated with KR. Red boxes indicate significantly upregulated genes, and blue boxes indicate significantly downregulated genes.


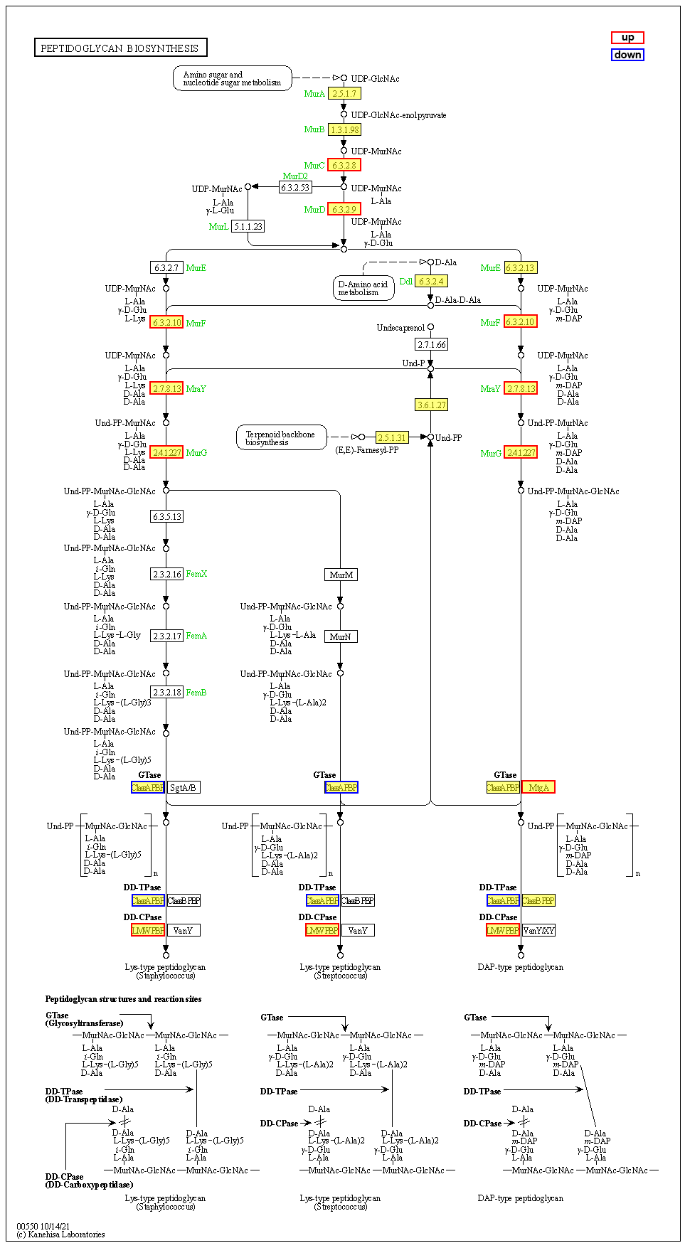


**Figure S12**. KEGG analysis of peptidoglycan biosynthesis in *E. coli* treated with KR. Red boxes indicate significantly upregulated genes, and blue boxes indicate significantly downregulated genes.


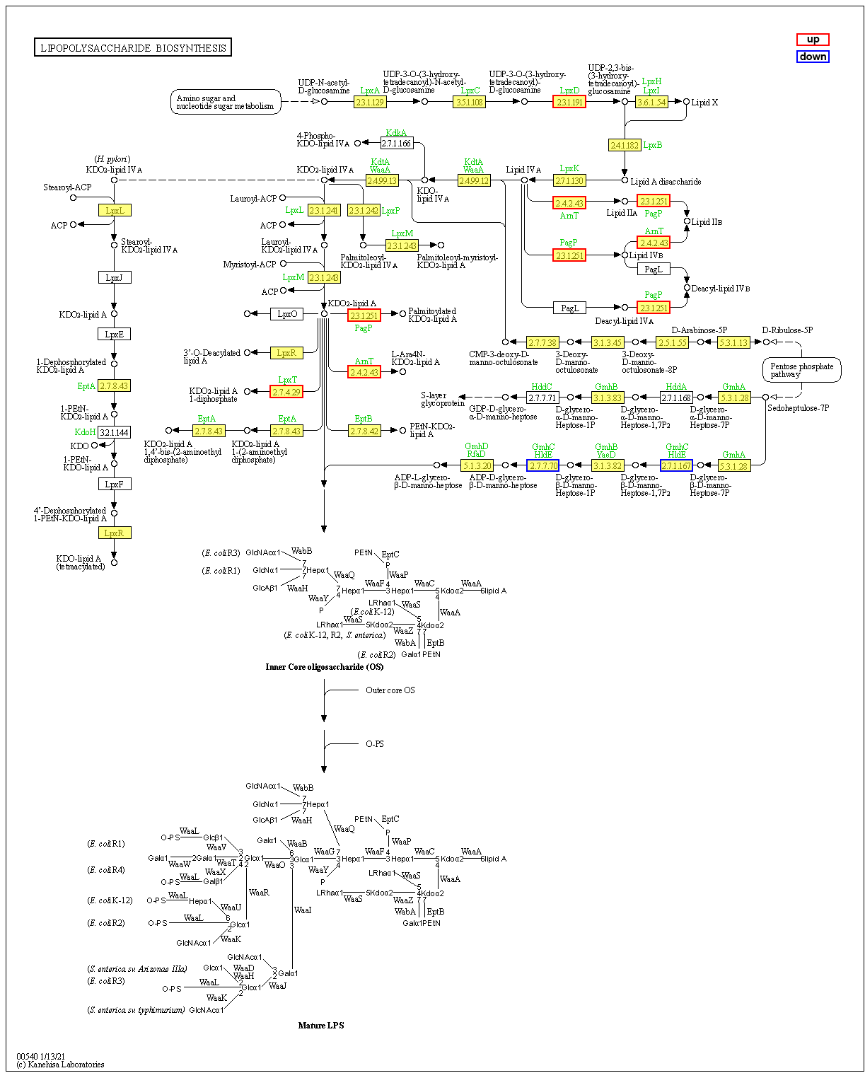


**Figure S13**. KEGG analysis of lipopolysaccharide biosynthesis in *E. coli* treated with KR. Red boxes indicate significantly upregulated genes, and blue boxes indicate significantly downregulated genes.


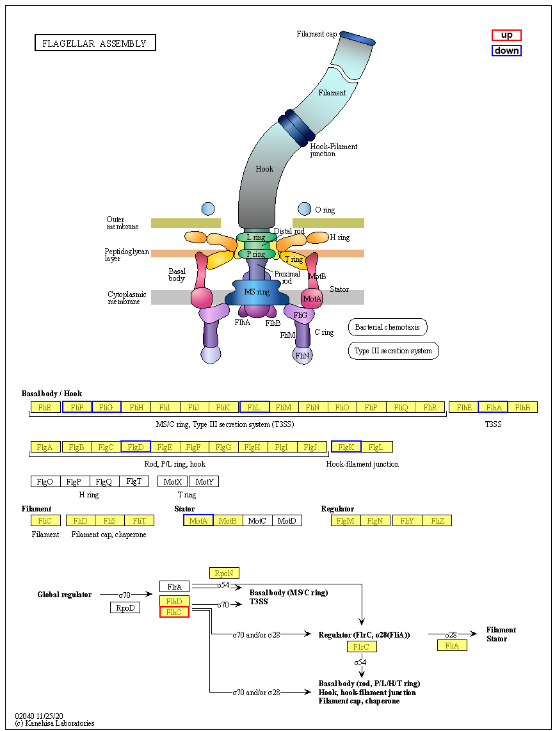


**Figure S14**. KEGG analysis of flagellar assembly in *E. coli* treated with KR. Red boxes indicate significantly upregulated genes, and blue boxes indicate significantly downregulated genes.


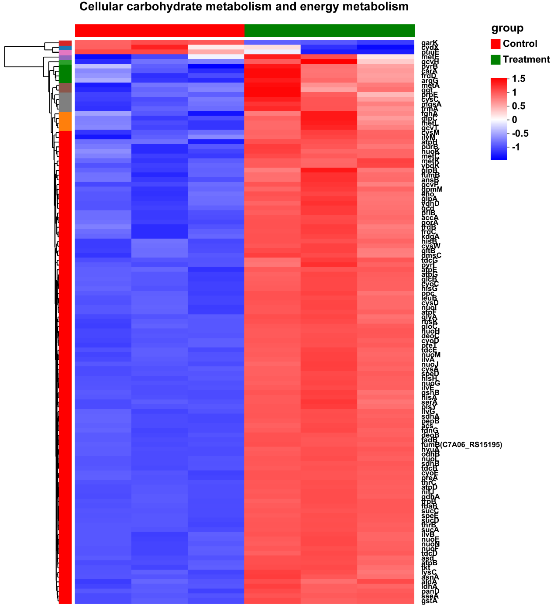


**Figure S15**. Heat map of cellular carbohydrate metabolism and energy metabolism-related differential genes expression. (P values < 0.05 and |log2(fold change)| > 1); blue indicated downregulated genes, while red indicated upregulated genes.


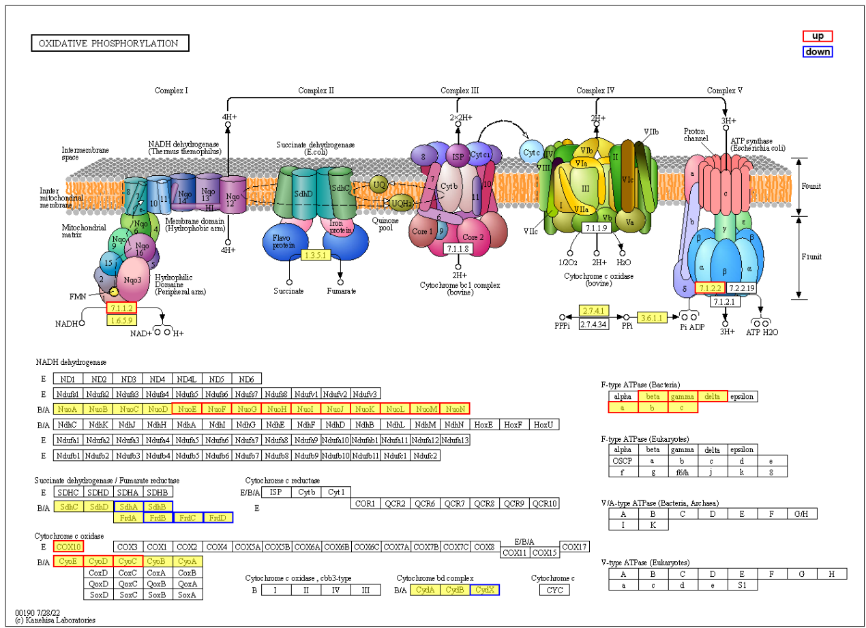


**Figure S16**. KEGG analysis of oxidative phosphorylation in *E. coli* treated with KR. Red boxes indicate significantly upregulated genes, and blue boxes indicate significantly downregulated genes.


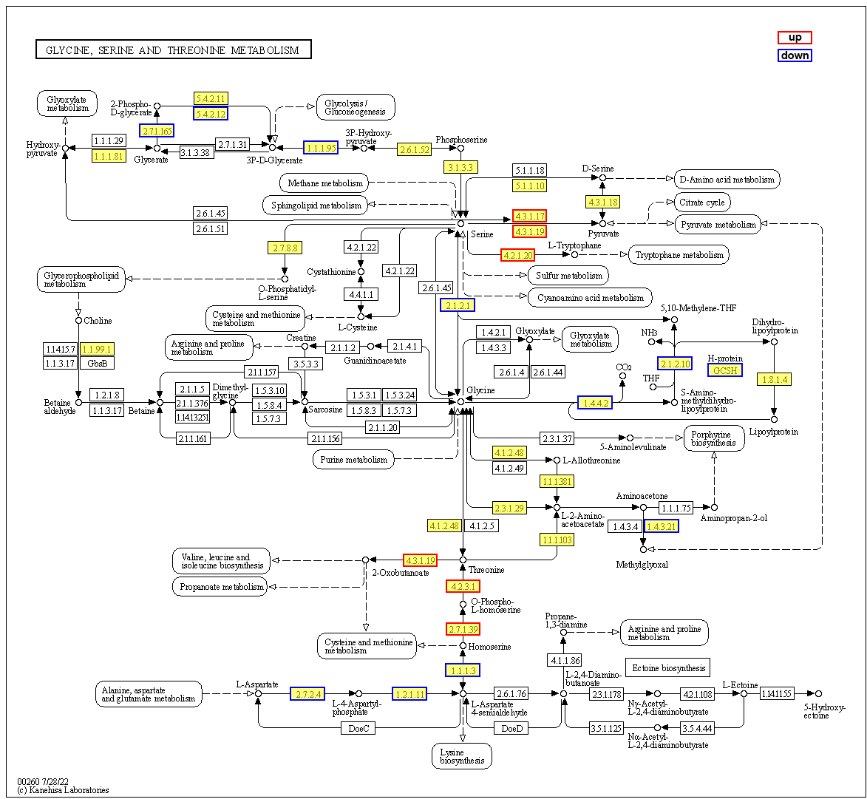


**Figure S17**. KEGG analysis of glycine, serine and threonine metabolism in *E. coli* treated with KR. Red boxes indicate significantly upregulated genes, and blue boxes indicate significantly downregulated genes.


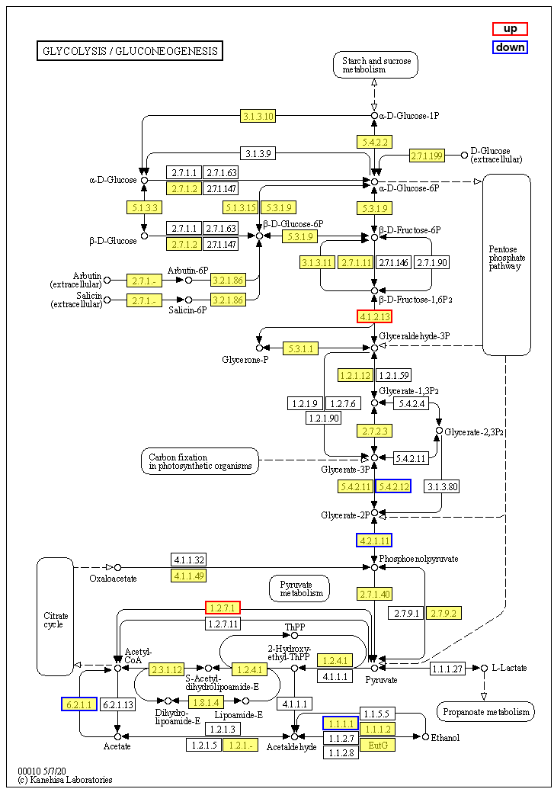


**Figure S18**. KEGG analysis of glycolysis/gluconeogenesis in *E. coli* treated with KR. Red boxes indicate significantly upregulated genes, and blue boxes indicate significantly downregulated genes.


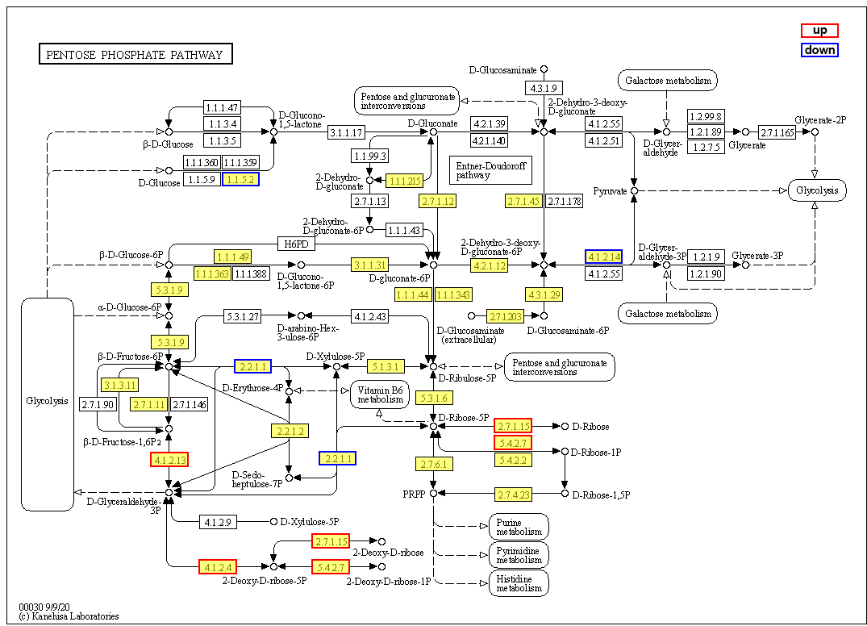


**Figure S19**. KEGG analysis of pentose phosphate pathway in *E. coli* treated with KR. Red boxes indicate significantly upregulated genes, and blue boxes indicate significantly downregulated genes.


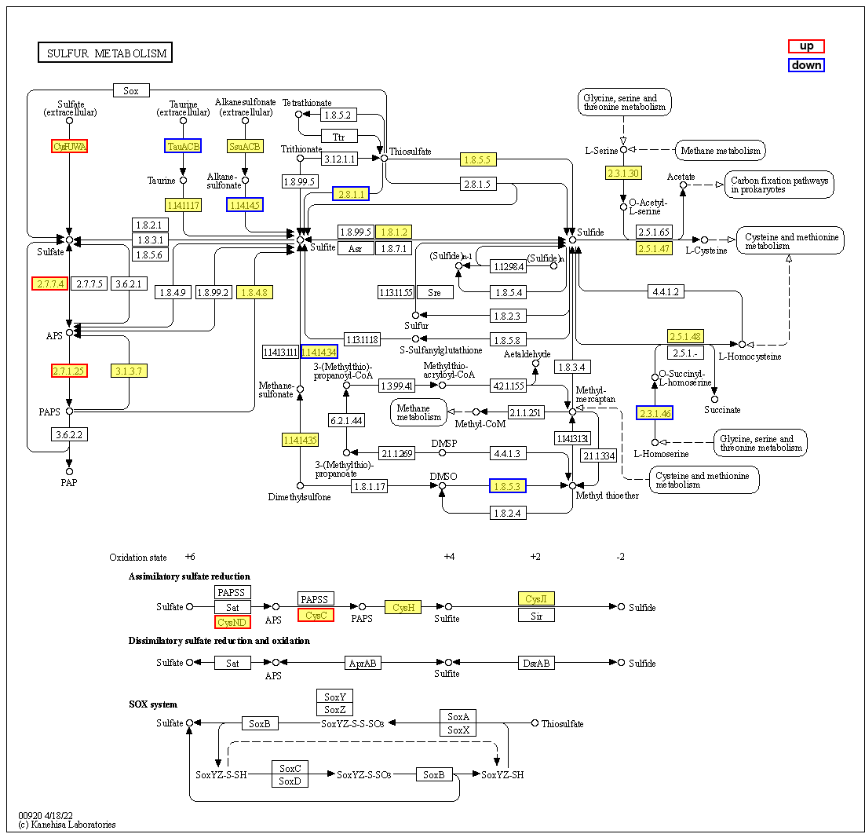


**Figure S20**. KEGG analysis of sulfur metabolism pathway in *E. coli* treated with KR. Red boxes indicate significantly upregulated genes, and blue boxes indicate significantly downregulated genes.


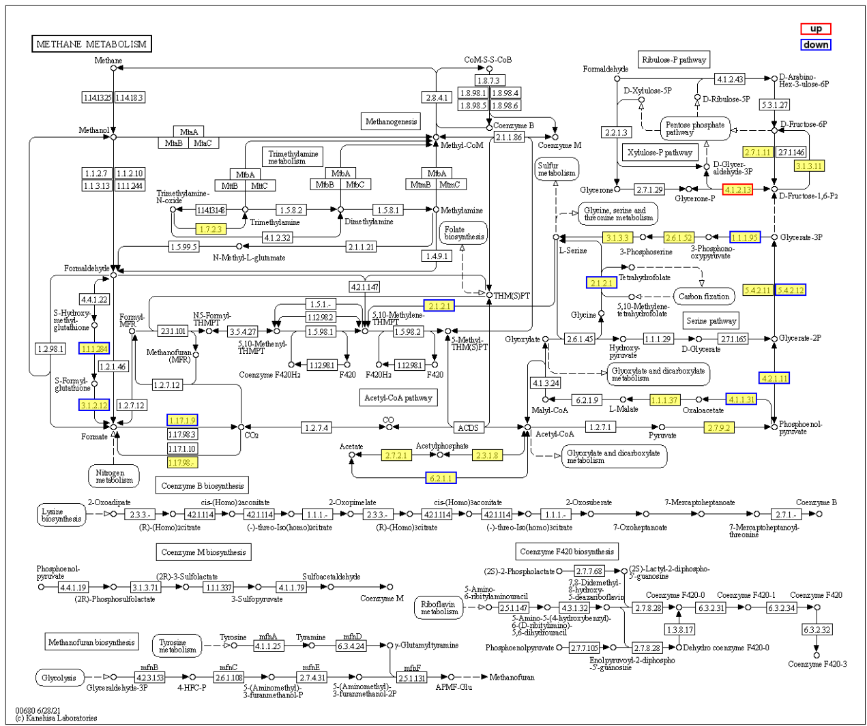


**Figure S21**. KEGG analysis of methane metabolism pathway in *E. coli* treated with KR. Red boxes indicate significantly upregulated genes, and blue boxes indicate significantly downregulated genes.


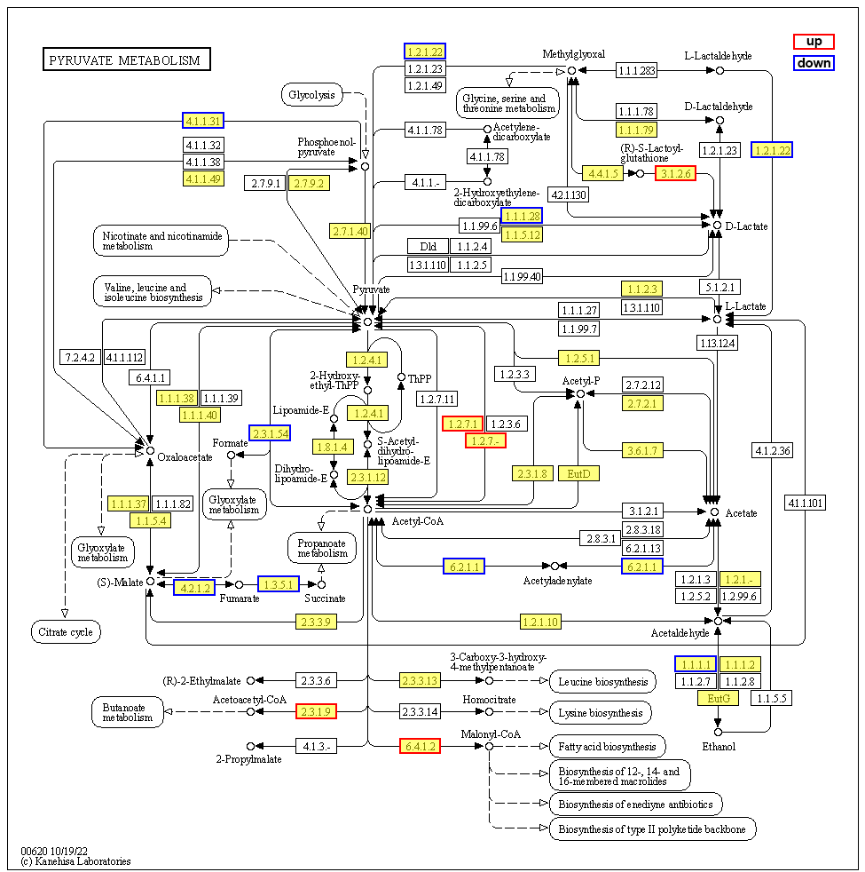


**Figure S22**. KEGG analysis of pyruvate metabolism pathway in *E. coli* treated with KR. Red boxes indicate significantly upregulated genes, and blue boxes indicate significantly downregulated genes.


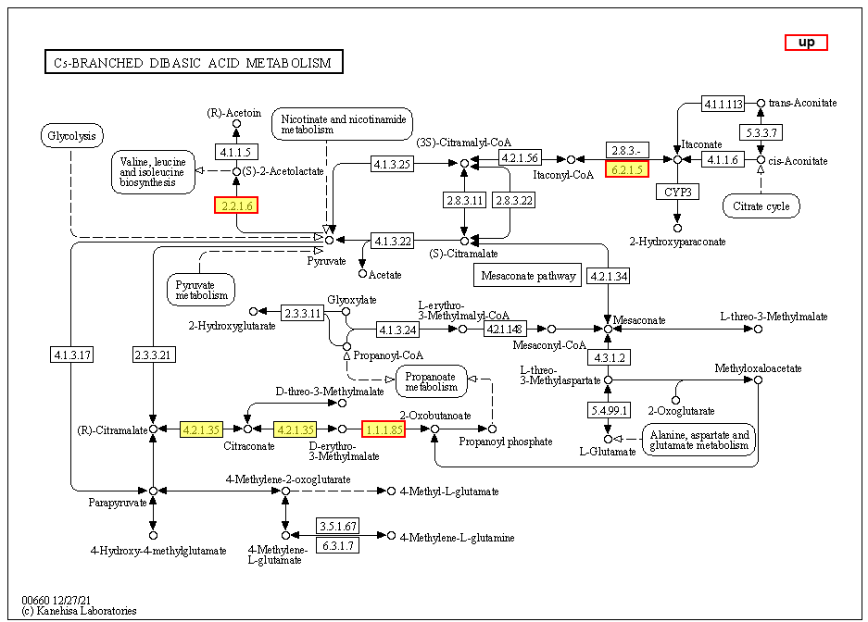


**Figure S23**. KEGG analysis of C5-branched dibasic acid metabolism in *E. coli* treated with KR. Red boxes indicate significantly upregulated genes, and blue boxes indicate significantly downregulated genes.


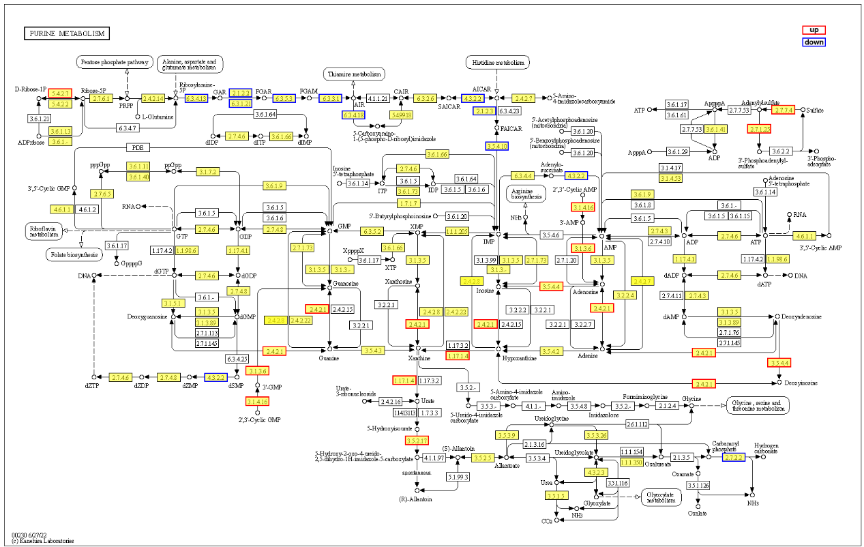


**Figure S24**. KEGG analysis of purine metabolism in *E. coli* treated with KR. Red boxes indicate significantly upregulated genes, and blue boxes indicate significantly downregulated genes.


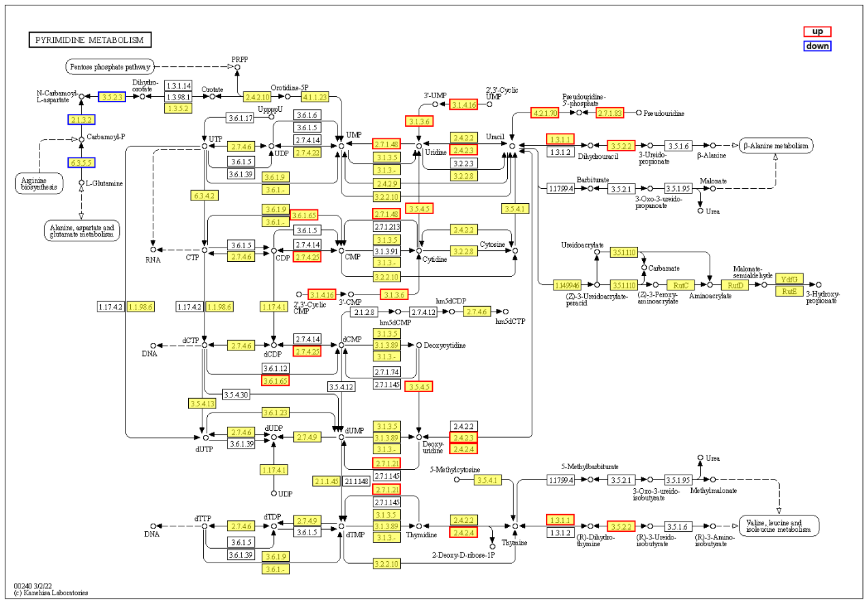


**Figure S25**. KEGG analysis of pyrimidine metabolism in *E. coli* treated with KR. Red boxes indicate significantly upregulated genes, and blue boxes indicate significantly downregulated genes.


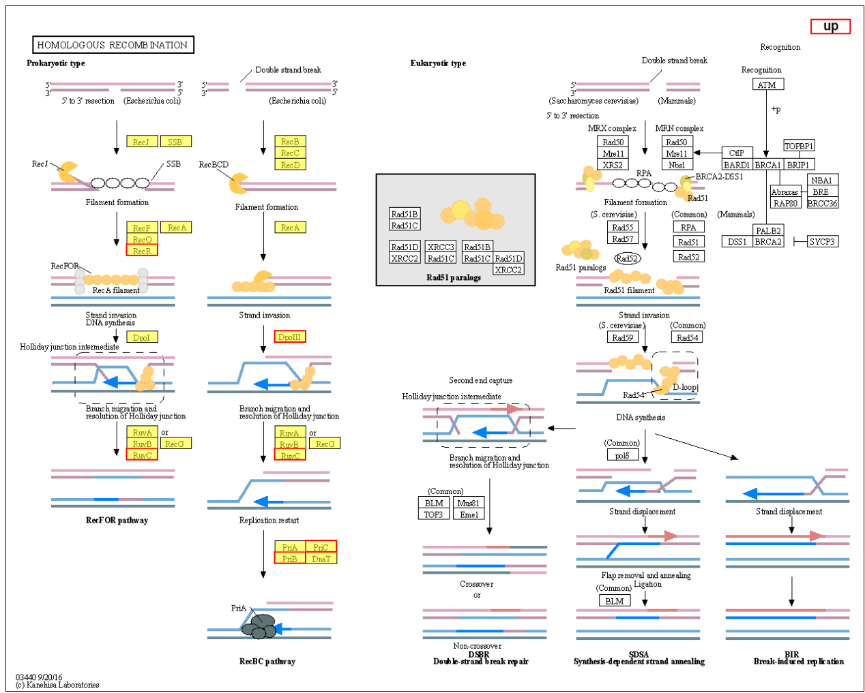


**Figure S26**. KEGG analysis of homologous recombination in *E. coli* treated with KR. Red boxes indicate significantly upregulated genes, and blue boxes indicate significantly downregulated genes.


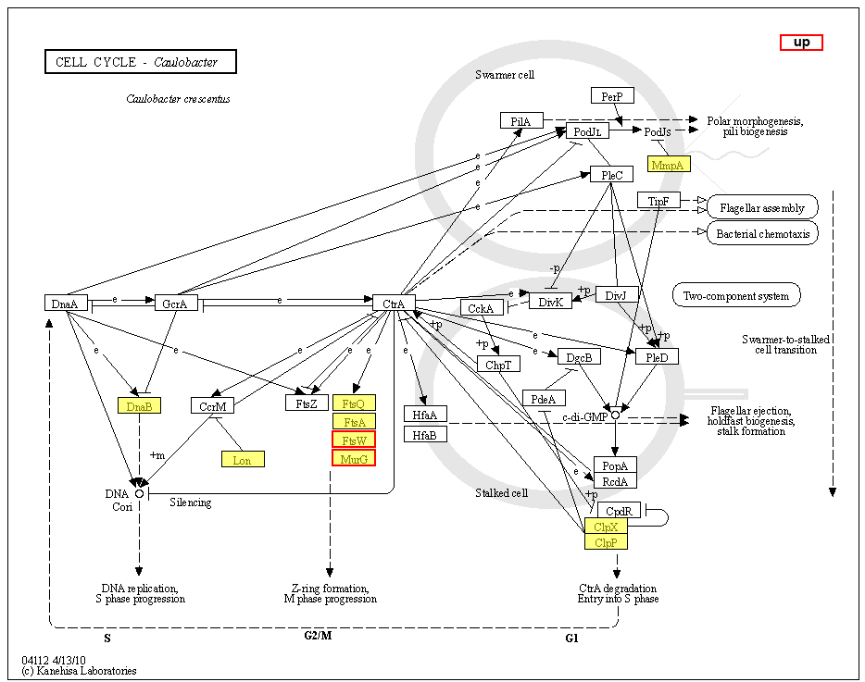


**Figure S27**. KEGG analysis of cell cycle in *E. coli* treated with KR. Red boxes indicate significantly upregulated genes, and blue boxes indicate significantly downregulated genes.


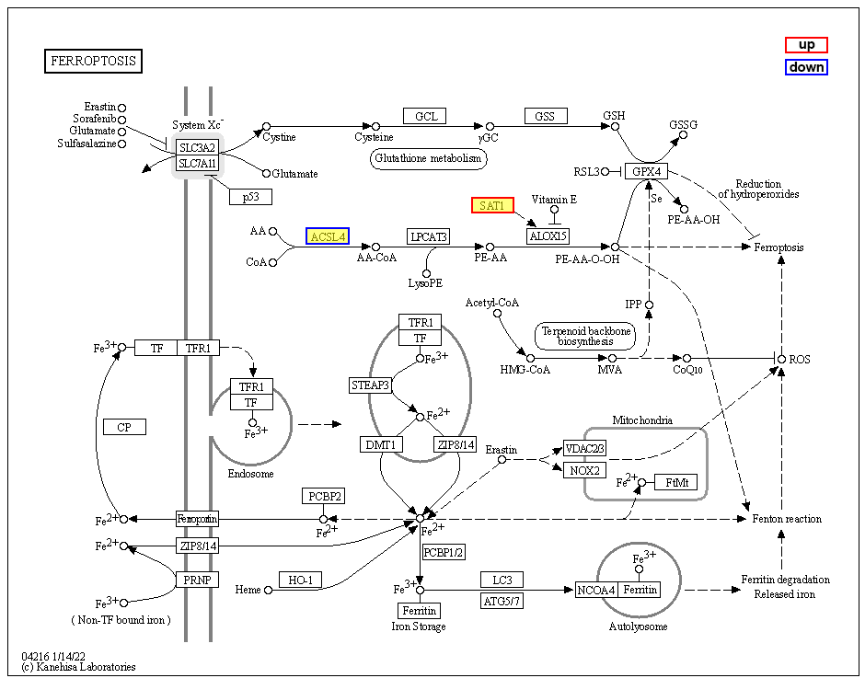


**Figure S28**. KEGG analysis of ferroptosis in *E. coli* treated with KR. Red boxes indicate significantly upregulated genes, and blue boxes indicate significantly downregulated genes.


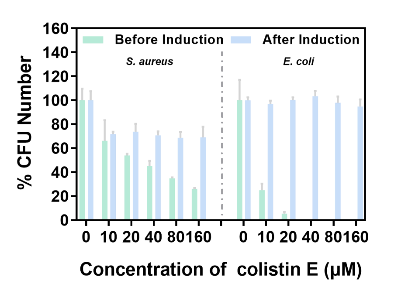


**Figure S29**. *S. aureus* and *E. coli* before and after drug resistance induction with Colistin E (4 μg mL^-1^).

**Table S1** The MICs (μM) of antibiotics commonly used in clinic against five bacteria

| Name | *S. aureus* | *E. coli* | *A. baumannii* | MDR-*E. coli* | MDR-*A. baumannii* |
| --- | --- | --- | --- | --- | --- |
| GSS | 128 | 256 | >256 | >256 | >256 |
| Colistin E | >256 | 256 | 32 | >256 | 128 |
